# Real-World Application of a Quantitative Systems Pharmacology (QSP) Model to Predict Potassium Concentrations from Electronic Health Records: A Pilot Case towards Prescribing Monitoring of Spironolactone

**DOI:** 10.3390/ph17081041

**Published:** 2024-08-07

**Authors:** Andreas D. Meid, Camilo Scherkl, Michael Metzner, David Czock, Hanna M. Seidling

**Affiliations:** 1 Internal Medicine IX: Department of Clinical Pharmacology and Pharmacoepidemiology, Medical Faculty Heidelberg/Heidelberg University Hospital, Heidelberg University, Im Neuenheimer Feld 410, 69120 Heidelberg, Germany; 2 Internal Medicine IX: Department of Clinical Pharmacology and Pharmacoepidemiology—Cooperation Unit Clinical Pharmacy, Medical Faculty Heidelberg/Heidelberg University Hospital, Heidelberg University, Im Neuenheimer Feld 410, 69120 Heidelberg, Germany

**Keywords:** potassium, kidney, spironolactone, quantitative systems pharmacology (QSP), electronic health records (EHR), sensitivity analysis, maximum a posteriori (MAP) (Bayesian) estimation

## Abstract

Quantitative systems pharmacology (QSP) models are rarely applied prospectively for decision-making in clinical practice. We therefore aimed to operationalize a QSP model for potas-sium homeostasis to predict potassium trajectories based on spironolactone administrations. For this purpose, we proposed a general workflow that was applied to electronic health records (EHR) from patients treated in a German tertiary care hospital. The workflow steps included model exploration, local and global sensitivity analyses (SA), identifiability analysis (IA) of model parameters, and specification of their inter-individual variability (IIV). Patient covariates, selected parameters, and IIV then defined prior information for the Bayesian a posteriori prediction of individual potassium trajectories of the following day. Following these steps, the successfully operationalized QSP model was interactively explored via a Shiny app. SA and IA yielded five influential and estimable parameters (extracellular fluid volume, hyperaldosteronism, mineral corticoid receptor abundance, potassium intake, sodium intake) for Bayesian prediction. The operationalized model was validated in nine pilot patients and showed satisfactory performance based on the (absolute) average fold error. This provides proof-of-principle for a Prescribing Monitoring of potassium concentrations in a hospital system, which could suggest preemptive clinical measures and therefore potentially avoid dangerous hyperkalemia or hypokalemia.

## 1. Introduction

Quantitative systems pharmacology (QSP) is a mechanistically oriented form of drug and disease modeling that provides an increasingly better understanding of disease processes and substantially supports drug development by evaluating intervention options [1,2,3,4]. Drug development is also increasingly supported by the integration of retrospective data from routine care such as electronic health records (EHR) [5,6,7]. Other applications of QSP models are scarce so far, as they are rarely applied prospectively to EHR, although they should have the capability to support decisions in routine care as well. EHR data can be obtained from electronic systems on a regular and timely basis. This includes, for example, drug administrations or relevant laboratory values, such as potassium [8], an important electrolyte that can lead to serious adverse events when levels are too high (i.e., hyperkalemia) or too low (i.e., hypokalemia). Therefore, a QSP model to predict potassium trajectories seems generally promising to test whether, how, and how well such models can be “repurposed” [4] for routine care decision support and how good their predictive performance is.

Clinically, plasma potassium level measurement is a favored method for monitoring hyperkalemia risk due to its directness, clinical relevance for complications (e.g., arrhythmia and muscle weakness), wide accessibility, and cost-effectiveness. Numerous risk predictions models have therefore been developed, for example, in the promising empirical prediction models by Eschmann et al. for continuous potassium monitoring [9] that have been implemented into their local clinical decision support to work with EHR in real time [10]. However, a QSP model is neither a rule-based decision-making tool nor an equation-based model in which the effects of risk factors (usually) enter additively. Instead, the QSP model describes a system with usually ordinary differential equations, for which covariate influences or drug influences can be considered. For EHR data, it is difficult to directly apply such QSP models due to various peculiarities. Usually, QSP models are developed, calibrated, and verified for plausibility with well-characterized reference data from highly controlled settings. These high demands also apply to potential validation, where the performance of a model is often determined in homogeneous data from similarly designed experiments [11,12]. Real-world EHR data, however, originate from a heterogeneous, largely uncontrolled setting. Here, not all relevant information may be known from available sources (e.g., procedure codes, unless they are manually retrieved from the records) [13]. Here, it remains questionable which information can be directly implemented in the model (as covariates) and which influential model parameters can be estimated from the available data. This can be more or less challenging and also depends on the complexity of the model (number of parameters and number of model compartments, the so-called state variables).

Considering plasma potassium, the QSP model of Maddah and Hallow precisely describes, with moderate complexity, the role of the kidneys and aldosterone feedback in maintaining potassium homeostasis [14]. In addition to the system part in physiological equilibrium, there is the pharmacological part, which influences this equilibrium. Among many influential substances, the present model focuses on spironolactone. Spironolactone and its metabolite canrenone are included via a classical pharmacokinetic model [15], in which canrenone levels are represented in a two-compartment model after spironolactone administration. The effect of canrenone concentration is then mediated via an Emax model and passed on to the system part. The effects are also well characterized in their dose dependence [16]. This model appears suitable as a starting point because it was developed based on solid literature knowledge on biological mechanisms and only a few parameters had to be estimated from experimental data for calibration. Nevertheless, it is not known to what extent the model parameters can be generalized outside the well-characterized conditions.

With this study, we intend to apply this established QSP model [14] in a routine setting of a university hospital to make model-based predictions about the further course of potassium concentrations. The predictive model performance of nine pilot patients could be investigated using drug regimens and laboratory values available from the newly introduced electronic patient chart. Such predictions could suggest preemptive clinical measures to avoid dangerous hyperkalemia or hypokalemia. Therfore, the model will be operationalized in such a way that it is able to use the accumulating clinical information using Bayesian inference. Because this will require numerous adaptations to specific conditions under routine care, our applied example can also be stimulating for future adaptations of QSP models in routine clinical settings. Thus, we aim to learn about the model itself and to gain experience for the implementation of QSP models in routine settings, which may have potential to support decisions in everyday therapeutic practice and thus improve patient care.

The remainder of this article is structured as follows: In Section 2, we present the results in a sequence as outlined in Appendix A. Immediately following, we discuss the implications of our results and provide an outlook on how such a project could be implemented as Prescribing Monitoring in routine care and how QSP modeling could reach the patient directly in terms of model-informed decision-making (Section 3). Details of the Materials and Methods can be found in Section 4 at the end.

## 2. Results

**Figure A1:**
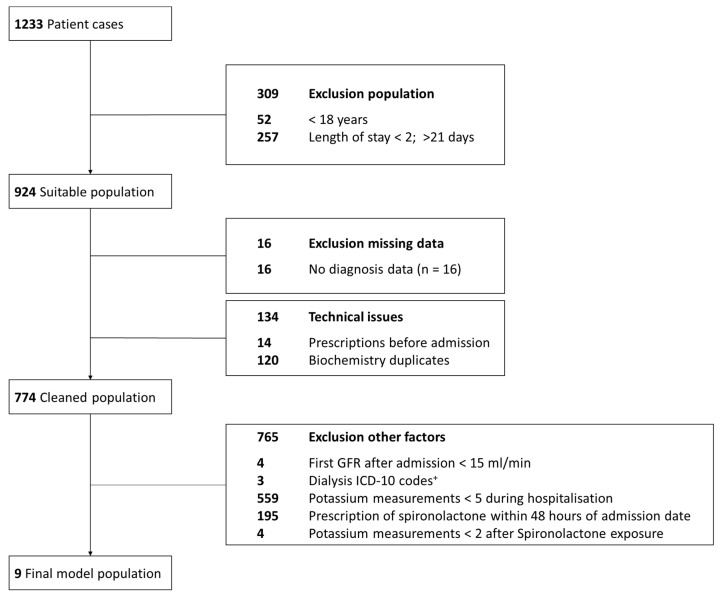
Patient flowchart. ICD-10: International Statistical Classification of Diseases and Related Health Problems 10th revision; GFR: Glomerular filtration rate; ICD-10 codes ^+^: N18.5, T82.4, Y60.2, Y61.2, Y62.2, Y84.1, Z49 *, Z99.2.

As a first step of our proposed workflow (Table A1), we obtained EHR data from nine patients newly initiating spironolactone in hospital with a median length of stay of 10.2 days (see Figure A1 and Table A2). In this pilot sample, four of them also received oral potassium supplementation in addition to a new spironolactone administration. Also, due to the strict selection of the pilot sample that is as homogeneous as possible (Figure A1), the included patients had no other risk factors for immediate potassium derailment (e.g., hemolytic anemia, renal impairment, diarrhea).

**Figure A2:**
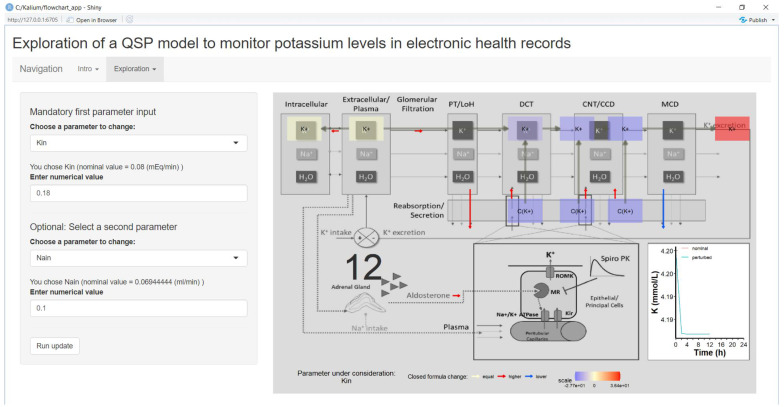
Live screenshot of the Shiny app. The Supplementary Material File S1, Figures S1-1–S1-15 provides animated gif produced with various inputs from Shiny app.

Second, the chosen original QSP model was operationalized for model examination. Via a Shiny app (Figure A2), in preparation for (global) sensitivity analyses, we changed up to two model parameters at once to examine the impact on state variables (compartments), transfer amounts between them, and the target potassium plasma level. For example, an increase in regular potassium intake increases the potassium level to a higher (steady-state) level, while the increasing concentration is also driving renal potassium secretion and ultimately the potassium excretion also increases with a higher aldosterone level (see animated figures in Supplementary Material File S1). Changing the glomerular filtration rate (GFR) in the model *alone* did not meaningfully influence potassium predictions, which is plausible because the mechanistically relevant single nephron GFR is a composite parameter (GFR divided by the number of nephrons). Therefore, any notable change would result primarily from the adjustment of both parameters. The associated nephron number must be adjusted proportionally if GFR is used as a covariate from patient data in the following. While sodium-related parameters appeared less relevant, the actually measured sodium plasma concentrations could inform the model’s normal level as a covariate.

**Figure A3:**
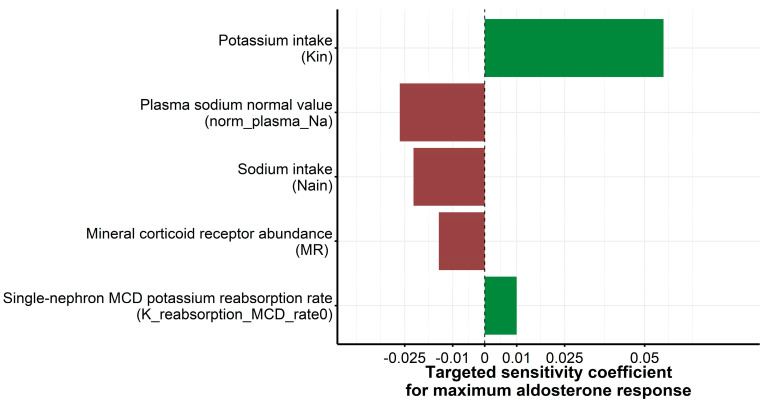
Targeted sensitivity analyses investigating parameter influence on maximum aldosterone response within 24 h (original model parameter names in brackets).

**Figure A4:**
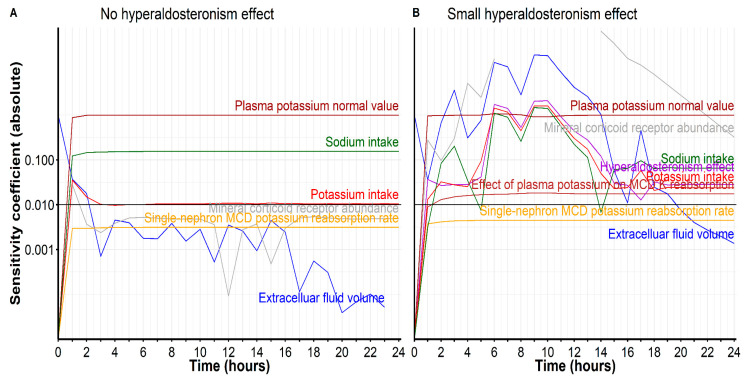
Local sensitivity analysis of potentially influential parameters in a situation with no hyperaldosteronism (**A**) or little hyperaldosteronism (**B**). The parameters studied included mineral corticoid receptor abundance (denoted as *MR* in the original model code), potassium intake (*Kin*), sodium intake (*Nain*), plasma potassium normal value (*norm_plasma_K*), plasma sodium normal value (*norm_plasma_Na*), extracelluar fluid volume (V_*ecf*), single-nephron medullary collecting duct (MCD) potassium reabsorption rate (*K_reabsorption_MCD_rate0*), plasma potassium effect on plasma aldosterone (*m_K_ALDO*), aldosterone effect on luminal potassium permeability (*Aldo_KSec_scale*), effect of plasma potassium on MCD potassium (K) reabsorption (*m_plasmaK_MCD*), and hyperaldosteronism effect (*hyperaldo_effect*) (display is limited to those parameters whose maximum value exceed 0.00001). The nominal (starting) value of the latter is set to zero in case of no hyperaldosteronism (**A**) or set to 0.1 in case of little hyperaldosteronism (**B**).

**Figure A5:**
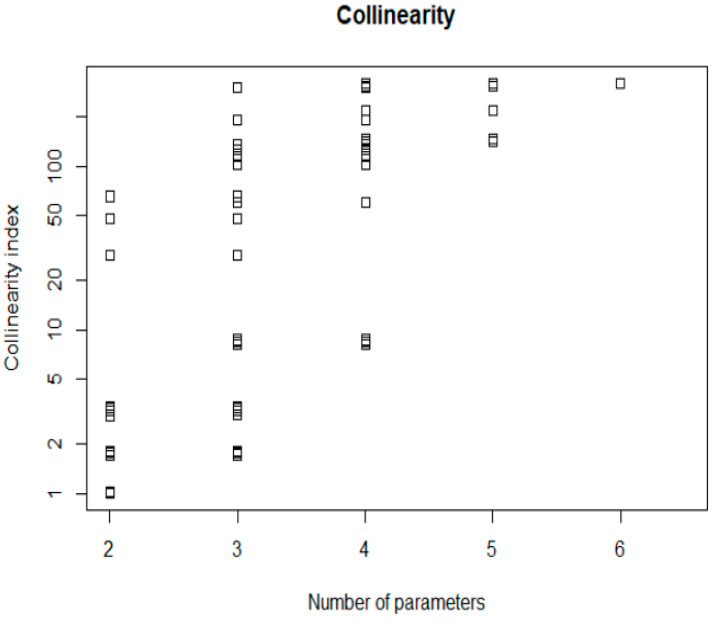
Collinearity indices γ obtained by local identifiability analysis of models with a varying number of model parameters estimated. Among the four-parameter models, either sodium intake or hyperaldosteronism effect could be included without exceeding the conventional collinearity limit of 15; models with mineral corticoid receptor abundance, potassium intake, extracellular fluid volume, and either sodium intake or hyperaldosteronism achieved collinearity indices γ of 8.42 or 8.12, respectively.

These impressions could be confirmed in local sensitivity analyses (SA), which is the third step. For example, the opposing influence of increased sodium or potassium intake on aldosterone levels was also apparent from SA (Figure A3). Local SA over time also showed that the influence of extracellular fluid volume is greatest immediately after perturbation of a parameter until equilibrium is reached (Figure A4). In the final model implementation later, the extracellular fluid volume was thus estimated only once at the beginning for each patient. It is also worth mentioning that the effect of mineral corticoid receptor abundance becomes relevant for a situation with at least mild hyperaldosteronism (Figure A4B). Among the parameters with only a minor influence (e.g., aldosterone normal concentration or rate constant for interstitial and intracellular potassium exchange), we kept the hyperaldosteronism effect as it could also indirectly represent a possible suppressive effect of ACE inhibitors (as a proxy). In an identifiability analysis, however, large collinearity indices γ resulted from models with both parameters for sodium intake and hyperaldosteronism included, which can be already expected from the model code, but should nevertheless be considered in case of problems with parameter estimation in later steps (Figure A5). We further chose to reduce the model complexity by fixing the plasma potassium normal value to the population-typical value.

**Figure 1:**
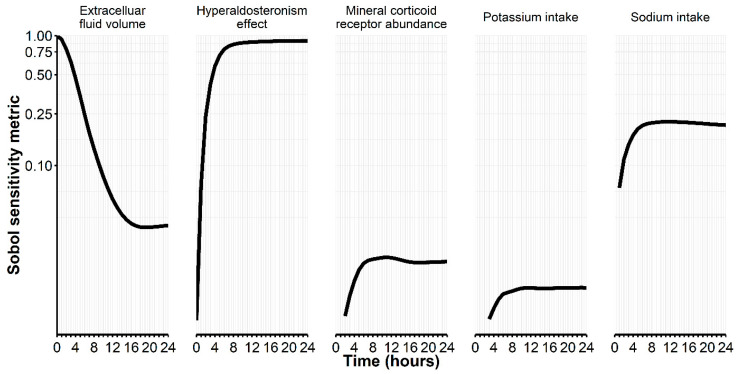
Global sensitivity analysis (SA) for pre-selected model parameters indicated by the Sobol sensitivity metric over 24 h. All parameters were allowed to fluctuate simultaneously—within physiologically plausible, predefined limits. If, for example, the upper limit for potassium intake is increased (e.g., in the case of supplementation), the sensitivity metric is much increased (where a value of 1 indicates highest impact and a value of 0.1 is still considered as sufficient impact by convention).

**Figure 2:**
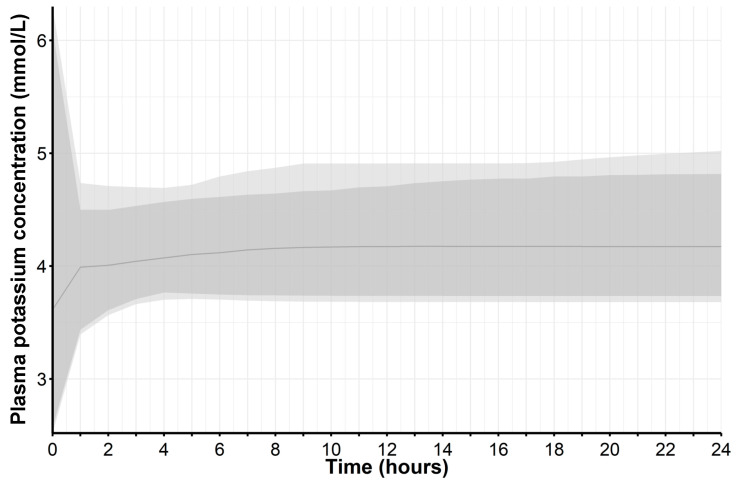
Uncertainty analysis for potassium trajectory simulation over 24 h. Simulations were based on a model with variations in parameters of potassium intake, sodium intake, mineral corticoid receptor abundance, extracellular fluid volume, and hyperaldosteronism effect. Monte Carlo sampling from physiological ranges of these parameters yielded simulated potassium values over time (thin line: median; dark-gray shaded area: 95% interval; light-gray shaded area: entire range within minimum and maximum values).

For the remaining parameters, a global SA largely confirmed their relevant influence within the model, although potassium intake and mineral corticoid receptor abundance remained below the conventional limit of 0.1 (Figure 1). Assuming physiological ranges in these parameters in an uncertainty analysis, we obtained clinically plausible simulated potassium trajectories from the model parameter distributions using Monte Carlo sampling (Figure 2). This uncertainty analysis thus showed that individual trajectories within a reasonable physiological range can result from variations in these parameters.

In order to obtain individual estimates for these parameters for the prediction of individual potassium courses, a range of their possible inter-individual variability (IIV) needs to the defined. This task belongs to the forth step and could be accomplished in historical data where IIV (reported as % coefficient of variation) were 5.2% for sodium intake, 18% for extracellular fluid volume, 36% for hyperaldosteronism effect, and 131% for mineral corticoid receptor abundance and potassium intake, respectively. Of note, in the subsequent application, the latter values were capped to a maximum of 80%.

**Figure 3:**
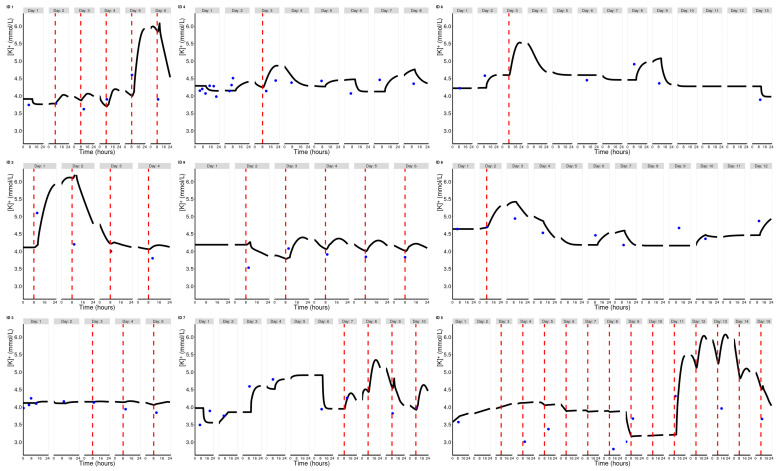
Observed potassium measurements (blue dots), spironolactone administrations (red dashed lines), and potassium trajectories predicted at midnight and at 11 am (black solid line). Of note, predicted baseline was derived from very first potassium measurement (defined as day zero).

Physiological mean values and IIV estimates were used as prior information for the Bayesian prediction of individual potassium trajectories in the fifth step. Because the Bayesian approach (which was conducted twice daily in our pilot case at midnight and at 11 am) can only consider previous information, the prediction pattern can lag behind, for example, and cannot capture random fluctuations. Figure 3 overlays the observed potassium measurements in the nine EHR cases with the model-predicted trajectories and spironolactone administrations. Overall, satisfactory performance measures were obtained for all patients in the EHR sample, including various subgroups (Table 1). While consistently within the acceptable range, we generally observed a tendency to overestimate.

## 3. Discussion

This pilot case shows how a repurposed QSP model could contribute to informed decision-making in everyday clinical practice. With increasing knowledge in the actual patient course, the model updates itself in a Bayesian approach to predict, in our case, the expected potassium course for the next 24 hours, which also takes planned drug administrations into account. Thus, the model prediction could give reason to preemptively modify potassium supplementation, to modify comedication affecting potassium concentrations, to reduce the spironolactone dose or, for safety, to arrange for additional laboratory measurements. This basic idea belongs to model-informed precision dosing [17,18,19], in which empirical (pharmacokinetic) models are becoming more and more established. When applied in the classical sense of a drug monitoring, drug concentrations as a surrogate for their effect are considered, while we could directly observe the pharmacodynamic response in terms of potassium measurements. Our use case presented here shows a proof-of-principle that this is also conceptually possible with mechanistic QSP models after being operationalized for this purpose.

Although our starting model was a mechanistic QSP model, its application is rather an empirical process when such a model is tailored to a specific patient case. How parameters are selected that are potentially influential and estimable may not always be straightforward to grasp from a clinical perspective. In terms of model content, this can be criticized and fragmentary evidence can be picked out that may argue against the operationalization made here. For example, potassium intake alone was not shown to be predictive of hyperkalemia [20], and although others factors contribute [21,22], it may clinically not seem reasonable that associated parameters (e.g., aldosterone response) are allowed to change very flexibly during the prediction time course. Nevertheless, for the empirical application, it is the predictive performance that counts.

While (random) fluctuations cannot always be explained clinically in heterogeneous everyday populations (e.g., errors in potassium quantification), for the predictions, there are numerous other clinical risk factors in routine care (not yet considered) so that the model can still be expanded. From a clinician’s perspective, our use-case model is actually a rather simple model (despite a certain complexity). It does not yet account for multiple possible risk factors for short-term potassium derailment, such as Addison’s disease, hemolytic anemia, gastrointestinal bleeding, rhabdomyolysis, tumor lysis syndrome, sickle-cell anemia, polyuria, acute/chronic diarrhea, and Cushing’s syndrome. These were not present in the selected pilot sample for this study. It was thus not possible to empirically assess the robustness of the model parameters across diverse clinical settings, incorporating various patient demographics and environmental conditions. Less robust results may be expected for some situations, such as those with severe renal insufficiency. Here, again, the application of the model in everyday clinical practice could provide data that could be considered as new evidence in revised models in the future.

We were fortunate to further evaluate an excellent, already published model for our purpose at hand [14]. This is not always the case, as often such models have to be created de novo and are usually not the result of an extension or modification of existing models [12]. Nevertheless, we were dependent on the already known limitations. The reference model of Maddah and Hallow [14] did not include humoral factors that alter aldosterone regulation, such as angiotensin II and adrenocorticotropic hormone [23]. They also assumed a direct effect of sodium intake on aldosterone secretion, a pragmatic decision, but one that is mechanistically thought to be mediated indirectly by changes in renin, which in turn are modulated by altered sodium intake. Overall, sodium homeostasis is only rudimentarily mapped in the current QSP model, but its integration is part of the group’s ongoing research.

**Figure A6:**
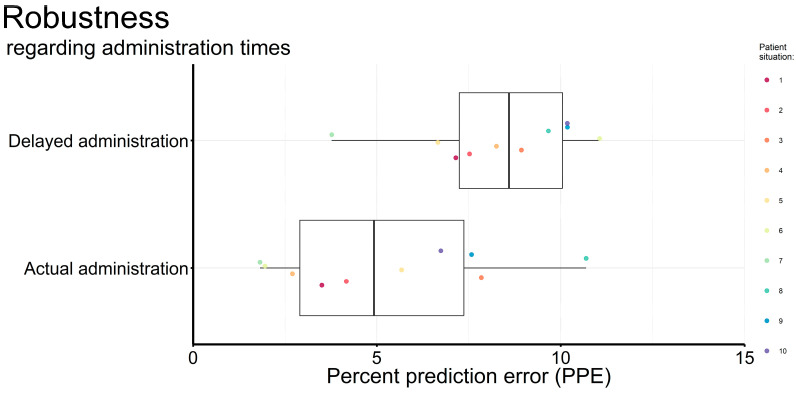
Robustness of the proposed approach with uncertainty in administration times. In a simulation experiment, ten baseline patients were randomly chosen in terms of their model parameters, covariates, and initial values of state variables to follow them up for five hospital days where spironolactone is administered on the last four days (50 mg scheduled at 8 am). Normally distributed random fluctuations (standard deviation, SD: 0.25) were added to the administration times, as well as for the five simulated potassium values (multiplied with mean 1 and SD of 0.075) recorded at 9 am. In addition to the “normal” scenario with these random fluctuations, a scenario with systematically late administration times (half an hour later than scheduled) was considered. Percent predictive error (PPE) was determined patient by patient and these values are shown here in the form of a boxplot.

**Figure A7:**
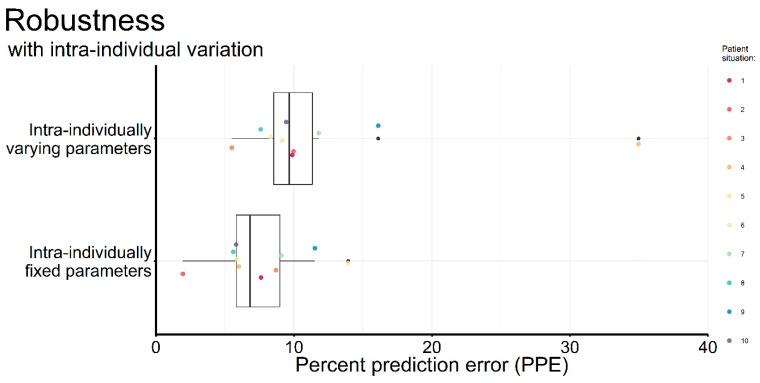
Robustness of the proposed approach with intra-individual variability in parameter estimates. In a simulation experiment, ten baseline patients were randomly chosen in terms of their model parameters, covariates, and initial values of state variables to follow them up for five hospital days where spironolactone is administered on the last four days (50 mg scheduled at 8 am, respectively). Normally distributed random fluctuations (multiplied with mean 1 and standard deviation, SD: 0.075) were added to the administration times, as well as for the five simulated potassium values (multiplied with mean 1 and SD of 0.1) recorded at 9 am. In addition to the “normal” scenario with these random fluctuations, a scenario with dynamically changing model parameters with each day was investigated (original parameter value multiplied with mean 1 and SD of 0.15. Percent predictive error (PPE) was determined patient by patient and these values are shown here in the form of a boxplot.

Limitations certainly lie in the database with regard to potential measurement errors or time deviations between the actual and planned spironolactone administrations. However, any efforts for data cleaning or plausibility checking would of course have also to carried out in real time for everyday use—one of many points that would still have to be addressed on the way to a possible implementation of such an approach. The nine pilot patients already provided a glimpse of the practical difficulties of our approach. If a potassium measurement immediately after spironolactone administration was unexpectedly high (e.g., ID 1—day 5, ID 2—day 1, ID 5—day 11), then the model tends to estimate the pharmacodynamic response to spironolactone individually high—so high that the estimator becomes biologically implausible and a clinically too high increase in potassium is predicted. This was particularly true for pilot patient 5. Triggered by the clinically implausible potassium increase, the IIV range was iteratively reduced here in order to reduce the amplitude to a clinically reasonable level. This may also be associated with convergence problems in the estimation process. In general, the intra-individual fluctuations (perhaps also circadian) sometimes appeared to be large. While these aspects should be investigated on a larger scale in future studies, the simulation experiments in Figure A6 and Figure A7 already suggest that both intra-individual variability and fluctuations in actual administration times may perturb the results obtained from our approach. One may speculate that the unexpectedly high predictions in the aforementioned patients may result from shifted spironolactone administration, which leads to an incorrect estimation of the response to spironolactone. Although these assessments are already informative given the current state of data, they cannot replace further investigations in the future, including (1) the determination of the robustness of model parameters across diverse clinical settings, (2) the validation of real-time data cleaning methods with a comprehensive set of clinical data, (3) the development of adaptive algorithms to adjust for intra-individual fluctuations in potassium levels in real-time, or even (4) conducting a comparative analysis between mechanistic-based and empirical models.

**Figure A8:**
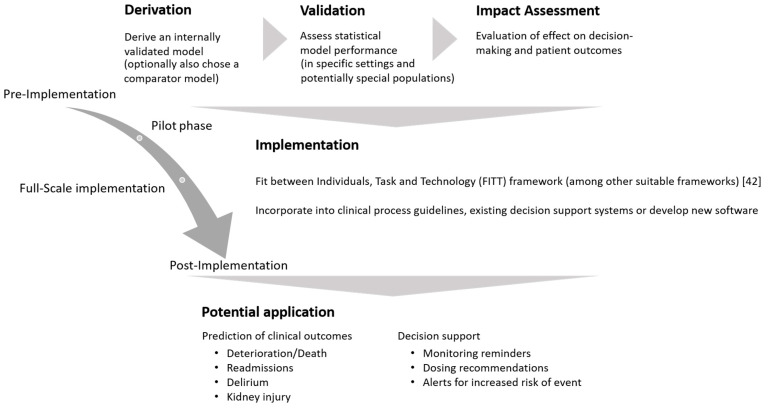
Steps required for implementing the QSP model in routine clinical settings. Crucial steps on the way to implementation are (model) validation in specific settings (and potentially special populations) and impact assessment preceding the actual implementation process. A strong argument would be a better performing model than available alternatives in a validation study. During pre-implementation, it is advisable to conduct stakeholder meetings, to design the alert interface and integrate with EHRs, and to develop training materials and simulation scenarios (in accordance with the FITT framework [42], among others). During the pilot phase, the alert system should be implemented in a small, controlled environment. Pilot users should be offered intensive training and support and feedback is to be gathered. At full-scale implementation, the system can be gradually expanded to other units or departments with continuously provided training and support, while also system performance and user feedback are to monitored regularly. Post-implementation then should include regular evaluations and user satisfaction surveys to facilitate iterative improvements based on collected data. Potential applications may refer to the clinical situation to monitor, while practical actions may include reminders for close patient monitoring, dosing recommendations, or alerts for increased risk of event.

**Figure A9:**
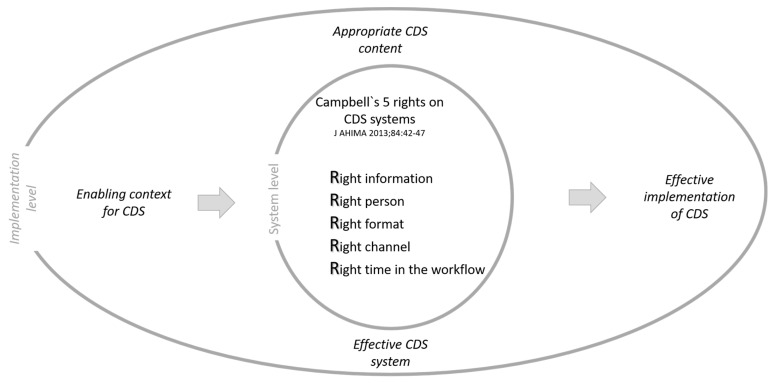
Framework-based overview on how alert acceptance of medication alerts can be increased in clinical routine. Based on the experience with rule-based alerts, optimizing the acceptance of model-based alerts in clinical routines requires a comprehensive framework that addresses technological, human, and organizational factors. Already in 2013 Robert Campbell suggested 5 rights for medication alerts, indicating that the right information has to be displayed to the right person, in the right format, through the right channel, at the right time in the workflow (Campbell R 2013). When it comes to implementation, the guides project offers more information on how the implementation process should be supported, including user training and monitoring (www.guidesproject.org). Italic: Items of the guides checklist on implementing clinical decision support systems (www.guidesproject.org). CDS = Clinical decision support.

We see crucial future research directions in the so-called Prescribing Monitoring, i.e., the continuous monitoring of the (medication) risks of individual patients during an inpatient stay. In the use of such a system, appropriate warnings could be issued when the medication is changed, or—as in our example—a prediction could be made for the following day and potential patients at risk could be indicated (e.g., before the ward round). But first, future work should compare how the mechanistic-based approach described here performs against purely empirical modeling approaches. Also, a combination of such different approaches appears promising. Such a combination could be implemented in such a way that a newly measured potassium value triggers a risk prediction with an empirical prediction model. This is because, in the current implementation, there was “only” a fixed forecast for the entire following day. However, if a measured value is now far away from this prediction, further potentially explanatory factors could be considered for an adjusted prediction. In this sense, it can be of course discussed whether the complete predicted potassium trajectory adds clinical value over the maximum potassium level over the next 24 h in decision-making. In empirical models, it might be sufficient to predict this future maximum level by using a wide variety of predictors as baseline predictors (e.g., GFR at admission), rolling predictors (e.g., maximum potassium level within a recent 12 h period), or growing predictors (e.g., cumulative spironolactone dose), perhaps supported by machine learning techniques. Whichever model is chosen as the basis for such prescribing monitoring, it must be validated in the same target group or under specific conditions, which is a mandatory prerequisite for the application of (QSP) models in the context of precision medicine [24]. Furthermore, before thinking about a possible implementation, numerous considerations (and possibly studies) are necessary on how to optimize the acceptance of such model-based alerts [13] by appropriate presentation and integration into the clinical routine. Figure A8 summarizes the potential steps required for implementing the QSP model in routine clinical settings and Figure A9 summarizes the aspects decisive for acceptance in routine care.

## 4. Materials and Methods

### 4.1. EHR Data Management

#### 4.1.1. Data Sources and Study Population

The pilot EHR data were routinely supplied. Patients who were admitted between 1 January and 31 March 2023 with a discharge date before 31 March were extracted from the digital curve from 17 wards at Heidelberg University Hospital, a 2500-bed tertiary care hospital. Information were issued from the local hospital information system (i.s.h.med^®^, Oracle Cerner, North Kansas City, MO, USA) that uses a Computerized Physician Order Entry (CPOE) system (i.s.h.med^®^ Smart Medication, Oracle Cerner, MO, USA) with an integrated clinical decision support system (CDSS) (AiD*Klinik*^®^, Dosing GmbH, Heidelberg, Germany). In this regard, local ethics vote (Ethics Committee of the Medical Faculty of Heidelberg University, ethical approval number: S-772/2022) governs the use of routine data to develop risk models regarding potassium trajectories.

#### 4.1.2. Data Preparation of Real-World EHR for Modeling and Simulation

For this pilot study, we considered only patients without potassium binders and without potassium infusion and included patients who initiated spironolactone during hospitalization and, if applicable, also received (oral) potassium supplementation. For administration schedules, we considered only complete cases with respect to spironolactone dosing schedules. Additional medication (e.g., diuretics or angiotensin converting enzyme inhibitors, ACE-I) was recorded for descriptive purposes to assess the predictive performance in these strata. To obtain a homogeneous sample for this pilot study, we further selected patients according to their potassium measurement (Figure A1). Other available laboratory measurements included estimated glomerular filtration rate (GFR) and sodium level. In summary, sodium plasma concentrations and GFR estimates were used as covariate information in the model. In most cases, the laboratory measurements are provided together, but it can happen that individual determinations were not made among the three laboratory values. In such cases, the “missing values” were replaced using the last-observation-carried-forward method (or, if necessary, with the standard value if it was the first potassium measurement).

### 4.2. Parameter Exploration via Shiny App

#### 4.2.1. Fundamental QSP Model

Our use-case model describes renal potassium filtration, reabsorption, and secretion along the nephron, and thereby incorporates potassium-aldosterone regulatory feedbacks to maintain whole body potassium balance, and allows for pharmacological modulation by the mineralocorticoid receptor antagonist (MRA) spironolactone [14]. The model is based on *N* nephrons as a functional unit, which includes the glomerulus and the tubule with its sections of proximal tubule and loop of Henle (PT/LoH), distal convoluted tubule (DCT), connecting tubule and cortical collecting duct (CNT/CCD), and medullary collecting duct (MCD). Based on mass conservation principles, differential equations determine the exchange of potassium quantities between model compartments (the state variables), i.e., intracellular and extracellular (plasma) amounts, tubule lumen amounts, tubule cell concentrations, and cumulative excretion. Model parameters encompass input of potassium and sodium, physiological normal concentrations, renal geometry, and function, as well as its alteration under hyperaldosteronism or MRA treatment.

#### 4.2.2. Shiny App

Following the original schematic of potassium regulation model (Figure 1 in [14]), we developed a Shiny app that allows for perturbing one and optionally, another model parameter. In the app, we show changes from the course with nominal standard parameters for compartments (as areas) and transfer quantities (as arrows) between them. These changes are shown in a red-blue color scaling as a percentage of the baseline value in 2 h intervals for 24 h; the plasma potassium concentration can be followed in an overlaid line chart (Figure A2, Supplementary Material File S1).

### 4.3. Parameter Sensitivity Analysis

#### 4.3.1. Local Sensitivity Analysis (SA) and Identifiability Analysis (IA)

Local SA of the QSP model-predicted potassium concentration *[K^+^]* over time was examined for a selection of parameters that was considered informative in an exploration of the Shiny app. We varied these parameters by 10% of the nominal (baseline) value to calculate the sensitivity coefficients (*S*) according to Equation (A1) (Appendix A). Generally, we assessed sensitivity functions over 24 hours. The magnitudes of the sensitivity metrics were used to rank the importance of parameters on the plasma potassium output with a liberal margin considering parameters with y > 0.01 as sensitive.

After initial model exploration, we investigated the potassium intake (denoted as *Kin* in the original model), plasma potassium normal value (*norm_plasma_K*), sodium intake (*Nain*), extracellular fluid volume (*V_ecf*), plasma potassium effect on plasma aldosterone (*m_K_ALDO*), aldosterone effect on luminal potassium permeability (*Aldo_KSec_scale*), effect of plasma potassium on MCD K reabsorption (*m_plasmaK_MCD*), single-nephron MCD potassium reabsorption rate (*K_reabsorption_MCD_rate0*), mineral corticoid receptor abundance (*MR*), and hyperaldosteronism effect (*hyperaldo_effect*). It is likely that local SA depends on the starting value of the parameters [11], which is why we studied two variants: A situation with standard aldosterone production and a situation with mild hyperaldosteronism. As a targeted sensitivity analysis for aldosterone response, the sensitivity metric function was adapted to focus on the maximum aldosterone concentration during the interval between 0 and 24 hours.

Local IA was conducted according to Brun et al. [25], simulating data from a model with perturbed parameter values generated from the nominal values plus a normally distributed error of mean zero and a coefficient of variation of 5%. We considered resulting collinearity indices γ as problematic and the parameter set as potentially poorly identifiable if γ > 15 [25].

#### 4.3.2. Global Sensitivity Analysis and Uncertainty Analysis

Among various methods to assess global sensitivity of model parameters, we chose the extended Fourier Amplitude Sensitivity Test (eFAST) to estimate the Sobol’ sensitivity measure with first- and total-order effects [26]. The eFAST method calculates the sensitivity measure through an analysis-of-variance-like decomposition of the function under analysis [27], which we applied with five replications and a simulated sample size of 1000. In particular, we studied global sensitivity while concomitantly perturbing parameters within a physiologically plausible range, i.e., potassium intake [0.073; 0.084] (mEq/min), sodium intake [0.01; 0.17] (mEq/min), extracellular fluid volume [10,000; 25,000] (mL), factors for mineral corticoid receptor abundance [0.8; 1.2], and hyperaldosteronism effect [−0.5; 0.5]. For uncertainty analysis, we applied Monte Carlo sampling from these parameter distributions to simulate potassium trajectories.

### 4.4. Parameter (Variability) Estimation

With these influential, potentially estimable parameters, a non-linear mixed-effects (NLME) model was fitted to estimate the IIV, which is later important for the IIV Ω in the Bayesian parameter estimation to predict individual potassium concentrations. For this purpose, we randomly selected 20 patient cases in advance of this study who initiated spironolactone during hospitalization, among whom 10 patients additionally received oral potassium supplementation. For NLME estimation, we used the first-order conditional estimation with interaction (FOCEi) algorithm with clinically orientated (upper and lower) boundaries to model parameters (see Section 4.3.2).

### 4.5. Bayesian Parameter Estimation to Predict Potassium Concentrations

#### 4.5.1. Estimation Setting of a (QSP) Prescribing Monitoring

We consider our use-case as a special case of so-called Prescribing Monitoring when a QSP model incorporating MRA administration is applied to monitor patient safety in routine care. Accordingly, we can monitor certain risks or physiological parameters such as potassium under the influence of drugs in a (hospital) system with a digital curve. Important are the attributes that the prediction (1) is longitudinal in the (2) individual patient situation, and that model predictions (3) are dynamic, i.e., the previous (risk) predictions inform future predictions. Thus, in a sense, there is a correlation of model parameters in the individual over time (autocorrelation), e.g., the extracellular volume estimated at day *d* influences the estimate at day *d +* 1.

Pragmatically, we have adopted a recently successful approach in therapeutic drug monitoring [28]. This goes back to loop control systems in anesthesia [29]. In our system, day 0 is the first day with potassium measurement for a particular patient. We use this first measurement to obtain an equilibrium in the QSP model for this patient. Therefore, we apply the QSP model in a Bayesian sense by combining priors from standard physiological values θ and variability Ω (IIV) with the observed potassium value at a long enough offset time to reach steady-state conditions (here: *t* = 3000 min) to obtain individual patient parameters η as priors for further steps. Initial values of state variables are also extracted from this equilibrium state. Empirical Bayesian estimates and initial (state) values are used to simulate for the upcoming day 1 and compare simulated values with any measured values for evaluation (see performance assessment below). This is conducted at two time splits: one at midnight and one at 11 am mimicking the time of the doctor’s visit. If another potassium measurement is taken on until one of those splits, then using the previously determined θ and IIV Ω as prior information for that day, the most likely individual parameters η are estimated. Generally, on day *d*, the individual parameter estimates from day *d* − 1 (or the same day at 11 am if a new observation is available until then) are used as a priori estimates ($\theta^{\prime }=\eta$), while the same IIV Ω is maintained [28]. If no measurement is made on a day, simulation alone is used and we proceed with corresponding final values on the following day.

#### 4.5.2. Performance Assessment

Predictive performance was assessed with metrics behind Equation (A5)–(A7) (Appendix A). Generally, we considered predictions as “satisfactory” if 0.8 ≤ AFE ≤ 1.25, as “acceptable” if 0.5 ≤ AFE < 0.8 or 1.25 < AFE ≤ 2, or as “poor” if AFE < 0.5 or AFE > 2. Applying the categorization accordingly to AAFE, we set the limits to AAFE ≤ 1.25, 1.25 < AFE ≤ 2, or AAFE > 2, respectively [30]. PPE was used for internal comparisons with lower values indicating better predictions.

### 4.6. Software

All analyses were implemented on an Ubuntu 20.04 server with R version 4.3.1 using the key packages *rxode2* and *nlmixr2* [31], *deSolve* [32], *FME* [29], *pksensi* [27], and *posologyr* [33].

## Figures and Tables

**Table 1:** Performance metrics.

Sample	Average Fold Error (AFE)	Absolute Average Fold Error (AAFE)	Percent Prediction Error (PPE) ^1^
**All** (*n* = 9)	1.06	1.19	7.3 [5.6; 9]
ACE inhibitor use during spironolactone (*n* = 4)	1.00	1.15	6.2 [2.9; 9.5]
Without potassium supplementation during spironolactone (*n* = 5)	1.07	1.19	7.1 [4.7; 9.4]
With potassium supplementation during spironolactone (*n* = 4)	1.04	1.20	7.7 [5.2; 10]
High-ceiling diureticsduring spironolactone(*n* = 5)	1.04	1.16	6.4 [4.4; 8.5]

^1^ Mean [in %] with 95% confidence interval.

## Data Availability

The clinical datasets presented in this article are not readily available because they originate from patient data specifically made available to the researchers for the particular purpose of this study. Further inquiries can be directed to the corresponding author.

## Supplementary Materials

The following supporting information can be downloaded at https://www.mdpi.com/article/10.3390/ph17081041/s1, Supplementary Material File S1: Figure S1-1–S1-15: Animated gif-figures from the Shiny App.

## Appendix A

The demands for the appropriate application of an already developed QSP model to a target population differ in some parts from the steps in the development of such a model [12,34]. When developing a QSP model, for example, impact analysis can inform about the appropriate parametrization, and structural identifiability analysis can provide insight into generating input data to estimate parameters [12]. Furthermore, potential modulators that are not the current focus of model development (but may be very important in clinical routine) can easily be disregarded. When applying a developed model to already existing data, though, the aforementioned techniques can help making informed guesses as to what parameters are worth considering an update. In this respect, we propose the following procedure aiming to directly incorporate as much empirical information as possible and to estimate only as many parameters as necessary (Table A1).

**Table A1:** Steps of the proposed workflow to operationalize the QSP model for its application in EHR data to inform decisions in clinical practice.

Step	Workflow Description	Key Questions to Answer
1	Data preparation of real-world EHR for Modeling and Simulation	How can the formats for laboratory measurements, medical history, and drug administration harmonized to allow model development and model application to predict future potassium trajectories?
2	Model exploration and parameter assessment	Which parameters are measurable, which are influential and clinically meaningful in EHR data?
3	Sensitivity: Impact analyses: local and global sensitivity [11,35] Identifiability [36]: parameter dependencies and collinearities	Which parameters are influential and estimable that allow flexible predictions when being estimated from real-world EHR data?
4	Variability in independent data Fixed-effects parameter θ estimation Inter-individual-variability (IIV) Ω determination	(a) Can parameters θ actually be estimated on a population level in real-world data? (b) How large is the expected IIV Ω and how can this expectation inform the design of the IIV matrix for the Bayesian a posteriori estimation (step 5)?
5	Bayesian a posteriori estimation [33] to predict future observations Setting determination: time points for estimation, forecasting period, … Performance assessment	How are prior information on individual parameters θ and their IIV Ω chosen for (updated) prediction in the longitudinal course of an inpatient stay?

Our ultimate goal is to predict the future potassium trajectories as part of a Prescribing Monitoring for individual patients in longitudinal EHR data under updating with newly available information, taking administrations with spironolactone (as well as other drugs and factors modulating potassium concentrations) into account. The Bayesian approach is suitable because a posteriori (conditional) distributions can be obtained from actual data and the current (a priori) parameter estimates the associated inter-individual variability, so that the distributions’ mode allows the individual best estimate of individual patient properties. Consequently, on the way to this goal, there are two requirements, namely: first, to know the influential parameters that can be reasonably estimated (sensitivity analysis, workflow step 3 in Table 1) and second, to have an informed guess on how much variability one can assume (workflow step 4 in Table A1).

Sensitivity analysis (SA) can be used to identify, especially from large models, those parameters that have the greatest influence on the results and therefore require more careful consideration. Such selection is also necessary because simultaneous optimization of too many parameters can lead to inadequate algorithm results [35]. Local SA examines changes in model results in response to individual parameter changes and can provide insight into which parameters should be examined in more advanced analyses. A sensitivity coefficient (*S*) after being parameter perturbation can be calculated for each parameter and included into a sensitivity matrix *S_i_*_j_ according to

(A1) $$\frac{\partial {\left[K^{+}\right]}_{i}}{\partial \theta_{j}}\cdot \frac{w_{\theta_{j}}}{w_{{\left[K^{+}\right]}_{i}}}$$

where ${\left[K^{+}\right]}_{i}$ is the potassium concentration as model output at time *i*, $\theta_{j}$ is a model parameter, $w_{{\left[K^{+}\right]}_{i}}$ is the (scaled) potassium concentration, and $w_{\theta_{j}}$ is the (scaled) parameter value [37].

Global SA examines all parameters (or those pre-selected by local SA, for example) simultaneously and provides insight into key parameters via a summary measure of induced changes in results. Many of such sensitivity indices exist [38] with the Sobol method as the most commonly and widely accepted measure [39].

Before parameters and their variability can be estimated, it is recommended to conduct (structural) identifiability analysis (IA) testing to determine whether the model structure and parameter estimates inferred from the known or assumed properties of a system are suitable and what parameters are uniquely identifiable by estimation procedures [12]. This can be achieved in local IA with simulated data from a model where random variation is introduced to predictions with nominal parameters [25]. Accordingly, a cost function estimates the sum of squared residuals and a linear dependence of parameter sets is approximated to provide an estimate of collinearity. Following Brun et al., collinearity γ is defined as

(A2) $$\gamma =\frac{1}{\sqrt{\min(\mathrm{EV}[{\hat{S}}^{T}\cdot \hat{S}]}}$$

where EV denotes the eigenvalue and with each element of parameter column in the sensitivity matrix $\hat{S}$.

(A3) $${\hat{S}}_{ij}=\frac{S_{ij}}{\sqrt{\sum_{j}S_{ij}^{2}}}$$

Consequently, the change in potassium level due to perturbation of one parameter can be compensated by a corresponding change in the other parameters by the fraction 1-1/γ [40].

SA and IA provide information on which parameter(s) (combinations) are likely to be successfully estimated. Non-linear mixed-effects (NLME) modelling is the method of choice for fitting empirical models to data while allowing for individual variation of each individual parameter estimate around its population mean. When, for a model parameter *j*, its estimate $\theta_{j}$ is considered to be log-normally distributed, then exponential random effects $\eta_{p}$ for an individual patient *p* describe inter-individual variability (IIV) to yield individual parameters $\theta_{p,j}$ according to

(A4) $$\theta_{p,j}=\theta_{j}\cdot e^{\eta_{p,j}}$$

where $\eta_{p}$ is a vector of normally distributed random effects (with length of all parameters *j*) with variance-covariance matrix Ω so that $\eta_{p}\sim N\left(0,\Omega \right)$.

For a Bayesian a posteriori estimation of individual parameters, the initial or recently estimated model parameters $\theta_{j}$ with their variance-covariance matrix Ω are used as prior information to estimate the individual random effects $\eta_{p}\;$ a posteriori, taking advantage of the observed data [34]. The individual parameter estimates obtained in this way can in turn be the starting point as prior information for subsequent estimation processes. Model validation of simulations with the parameters thus obtained can rely on the following metrics to assess the predictive performance of the approach comparing predicted values (*Pred*) to observed values (*Obs*):

(A5) $$\mathrm{Average}\;\mathrm{fold}\;\mathrm{error}\;(\mathrm{AFE}):\;AFE=10^{\frac{1}{n}\sum log\frac{{Pred}_{p,i}}{{Obs}_{p,i}}}$$

(A6) $$\mathrm{Absolute}\;\mathrm{average}\;\mathrm{fold}\;\mathrm{error}\;(\mathrm{AAFE}):\;AFE=10^{\frac{1}{n}\sum \left|log\frac{{Pred}_{p,i}}{{Obs}_{p,i}}\right|}$$

(A7) $$\mathrm{Percent}\;\mathrm{prediction}\;\mathrm{error}\;(\mathrm{PPE}):\;PPE\;\left(\%\right)=Mean\left(\left|\frac{{Pred}_{p,i}-{Obs}_{p,i}}{{Obs}_{p,i}}\right|\cdot 100\right)$$

where *n* is the number of observations from patients *p* at times *i*.

## Appendix B

**Table A2:** Patient characteristics.

Subject Characteristic	1	2	3	4	5	6	7	8	9	Overall (N = 9) Mean (±SD), Median [Min, Max] or %
Age [years]	74	88	57	70	56	57	59	83	66	68 (±12)
Sex (male = 1)	male	female	male	male	female	male	male	male	male	Female: 22%
Weight [kg]	85.0	54.0	92.0	104.0	85.0	93.0	87.0	89.0	90.0	87 (±14)
Length of stay [days]	6.4	5.8	5.9	9.0	18.8	13.0	9.7	14.1	8.9	10.2 (±4.4)
**Baseline laboratory measurements**										
eGFR (CKD-EPI) [ml/min/1.73 m^2^]	69	73	59	33	103	91	101	27	89	72 (±28)
Sodium [mmol/L]	142	143	135	143	133	142	137	140	138	139.22 (±3.67)
Potassium [mmol/L]	4.13	4.07	4.09	4.13	3.56	4.12	3.92	4.86	4.18	4.12 (±0.34)
Spironolactone dose	25	25	50/100	25	100	25	25	50	25	25 [25, 100]
**Co-medication during spironolactone administration**										
ACE inhibitors ^1^ [y/n]	no	yes	no	no	no	yes	no	yes	yes	44%
Angiotensin receptor blocker ^2^ [y/n]	yes	no	no	no	no	no	no	no	no	11%
High ceiling diuretics ^3^ [y/n]	no	no	yes	no	yes	no	yes	yes	yes	56%
Low ceiling diuretics ^4^ [y/n]	no	yes	no	no	no	no	no	no	no	11%
Oral potassium additives ^5^ [y/n]	no	no	no	no	yes	no	yes	yes	yes	44%
**Selected comorbidities (Elixhauser** [41])										
Congestive heart failure [y/n]	yes	no	no	yes	no	yes	yes	yes	yes	67%
Cardiac arrhythmias [y/n]	no	yes	no	yes	yes	yes	yes	no	no	56%
Hypertension, uncomplicated [y/n]	no	no	no	yes	no	no	yes	yes	yes	44%
Diabetes, uncomplicated [y/n]	no	no	no	yes	no	no	no	no	no	11%
Hypothyroidism [y/n]	no	no	yes	no	yes	no	no	no	no	22%
Liver disease [y/n]	no	no	yes	no	yes	no	no	no	no	22%
Coagulopathy [y/n]	no	no	yes	no	no	no	no	no	no	11%
Fluid and electrolyte disorders [y/n]	no	no	no	yes	yes	no	no	no	no	22%
Alcohol abuse [y/n]	no	no	yes	no	yes	no	no	no	no	22%
Elixhauser total score (unweighted)	1.00	1.00	4.00	5.00	6.00	2.00	3.00	2.00	2.00	2.00 [1.00, 6.00]

ACE: Angiotensin Converting Enzyme; CKD-EPI: Chronic Kidney Disease Epidemiology Collaboration; eGFR: estimated Glomerular Filtration Rate ATC-codes: ^1^ C09A, C09B; ^2^ C09C, C09D; ^3^ C03C; ^4^ C03A, C03B; ^5^ A12BA.

## Appendix C

Operationalized R code (rxode2 format transported from the RxODE code from the original QSP model developed by Maddah and Hallow 2022 [14].
