# Supplementary figures and images for: Real-World Application of a Quantitative Systems Pharmacology (QSP) Model to Predict Potassium Concentrations from Electronic Health Records: A Pilot Case towards Prescribing Monitoring of Spironolactone
Source: Pharmaceuticals (Basel). 2024 Aug 7;17(8):1041. doi: 10.3390/ph17081041 (PMC11357243; doi:10.3390/ph17081041)

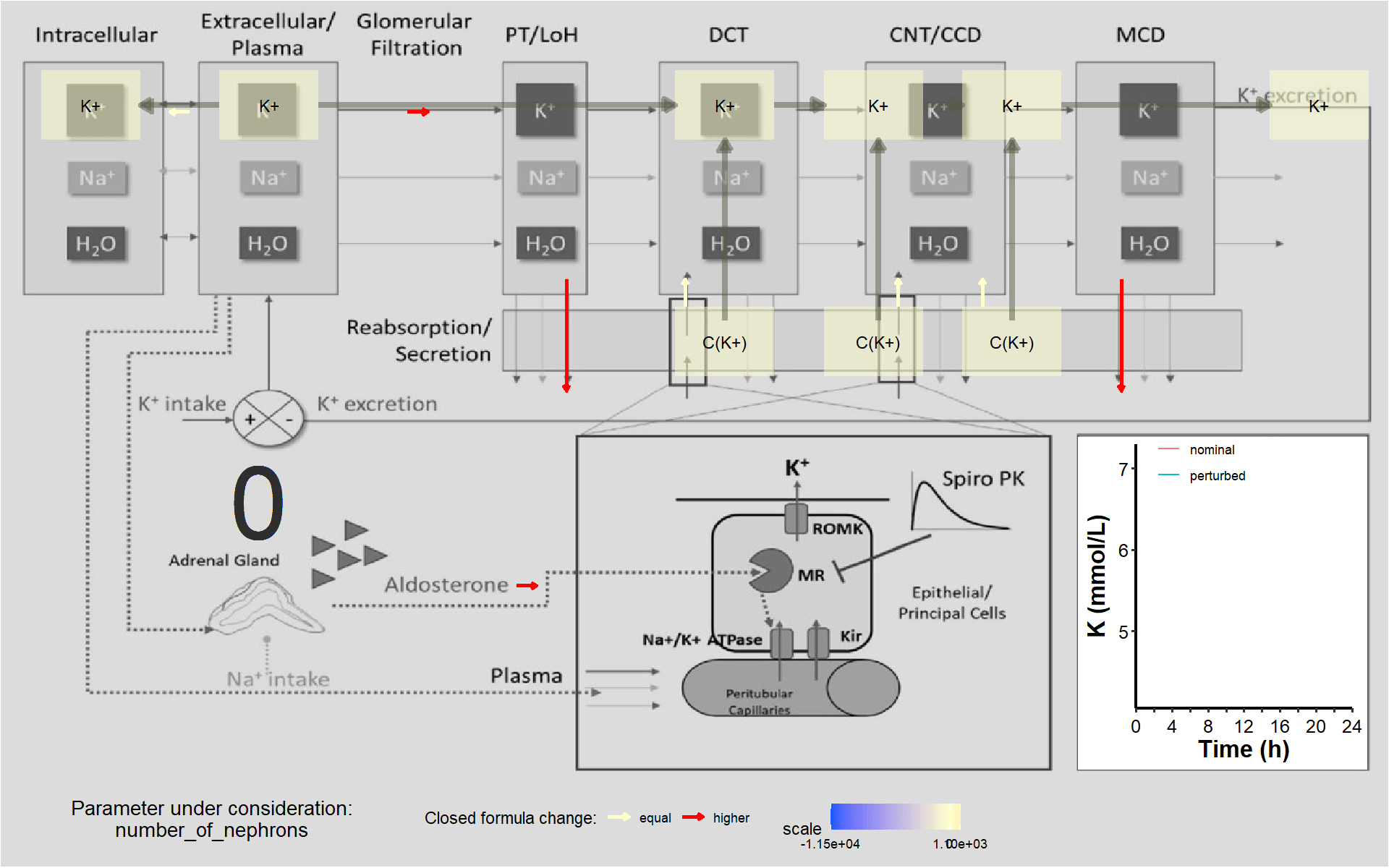

Supplement: Supplementary file 1 [file pharmaceuticals-17-01041-s001.zip › Figure S1-10_number_of_nephrons_20000_Nain_0.03.gif]

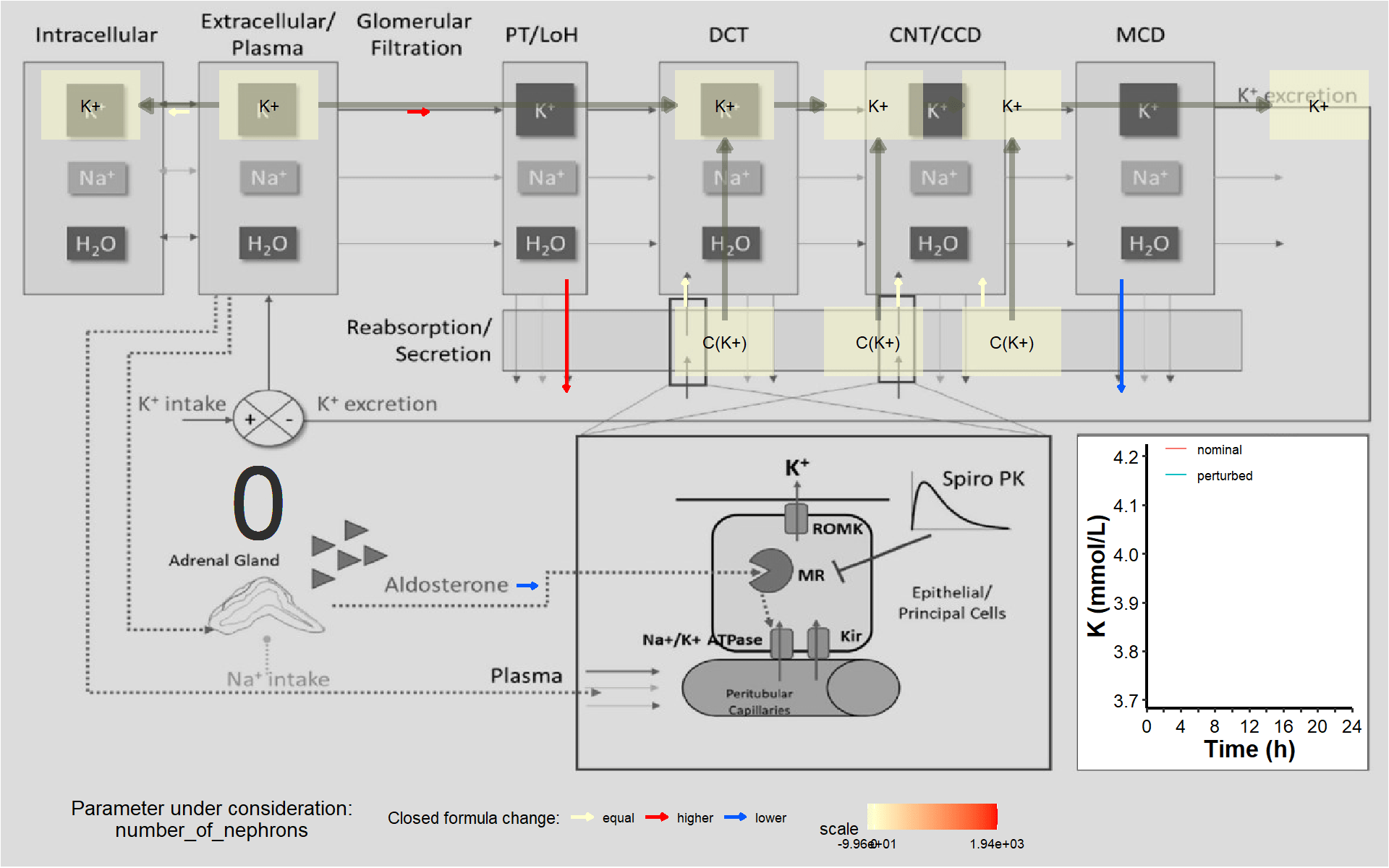

Supplement: Supplementary file 1 [file pharmaceuticals-17-01041-s001.zip › Figure S1-11_number_of_nephrons_20000_Nain_0.17.gif]

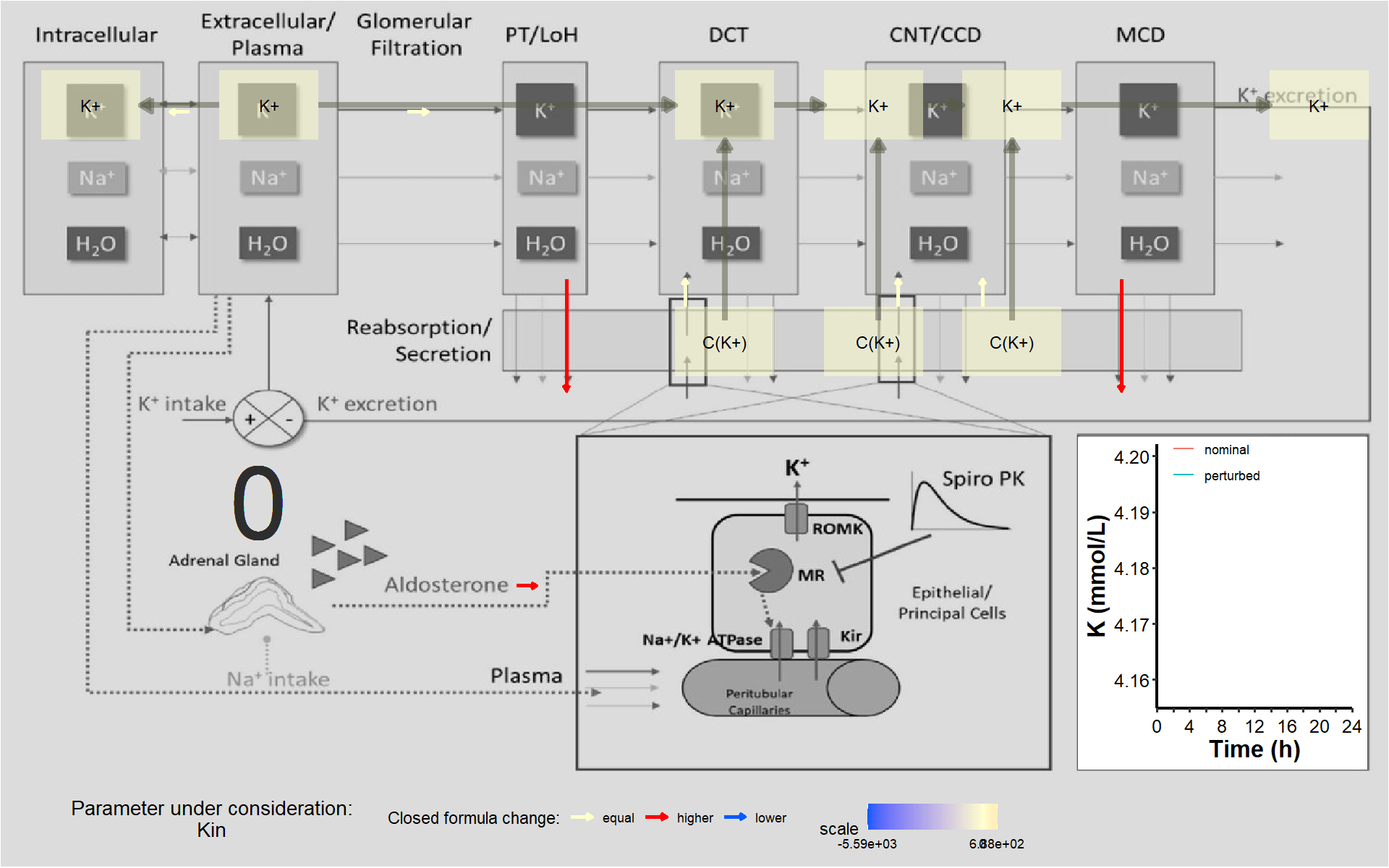

Supplement: Supplementary file 1 [file pharmaceuticals-17-01041-s001.zip › Figure S1-12_Kin_0.04_Nain_0.03.gif]

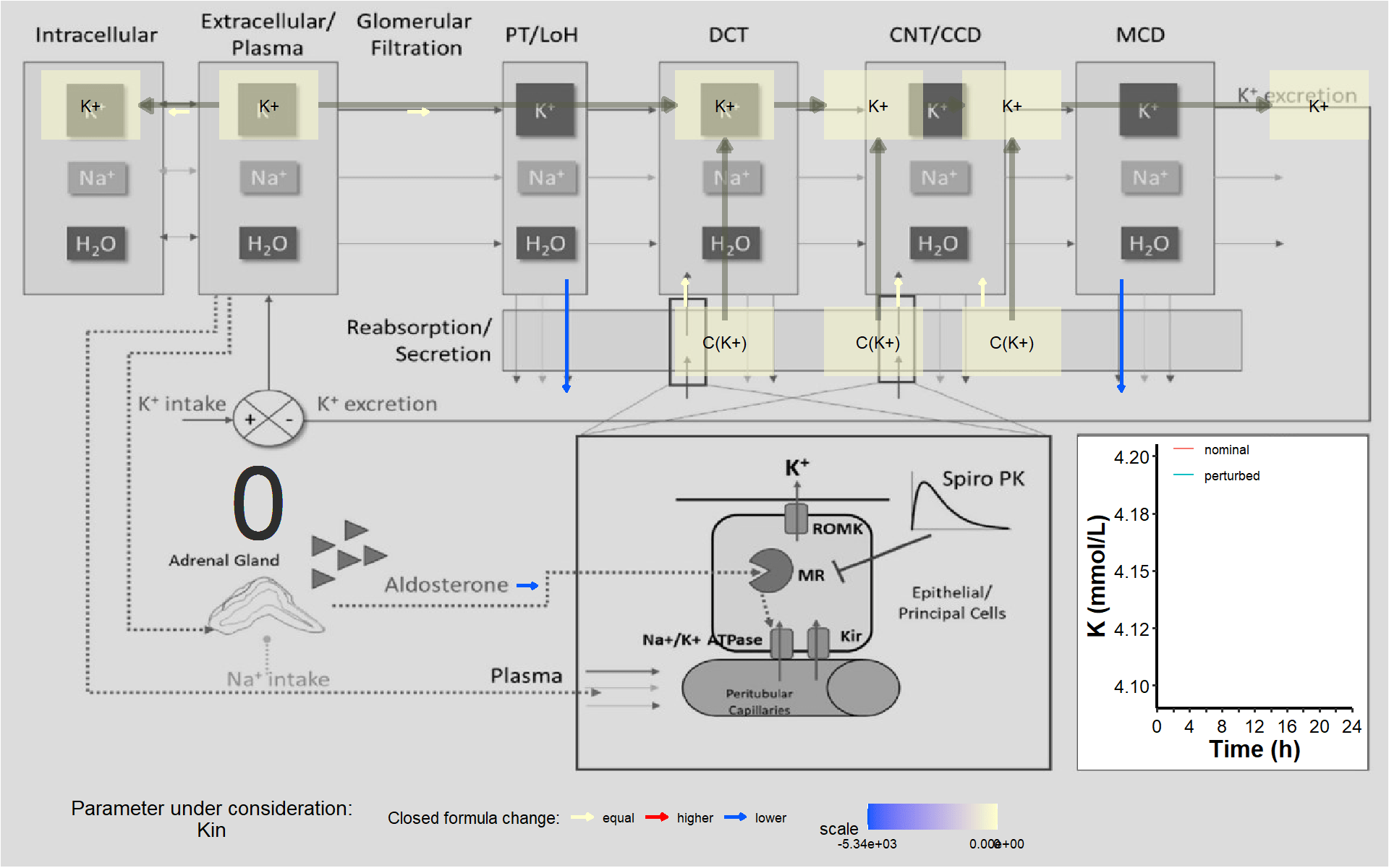

Supplement: Supplementary file 1 [file pharmaceuticals-17-01041-s001.zip › Figure S1-13_Kin_0.04_Nain_0.17.gif]

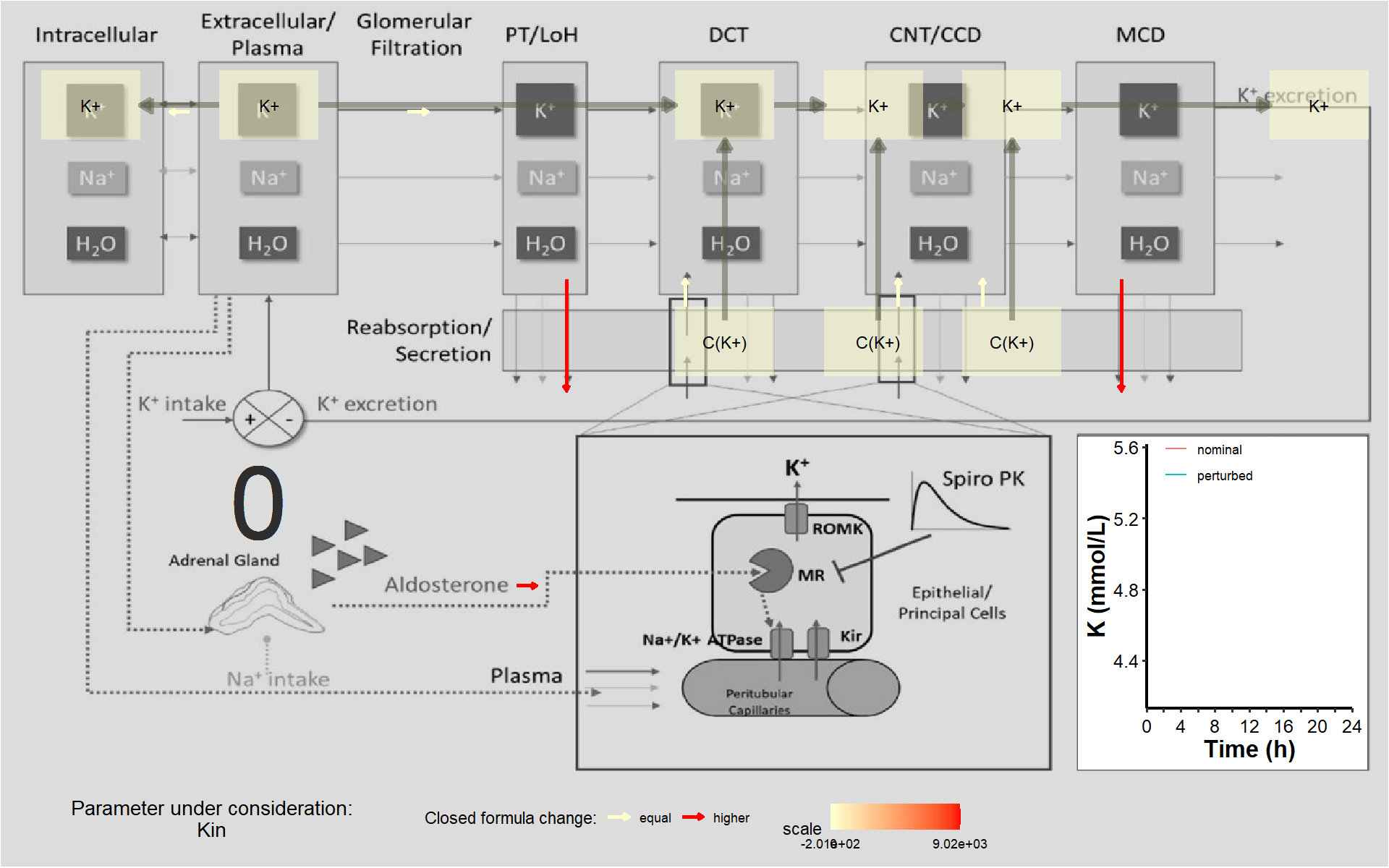

Supplement: Supplementary file 1 [file pharmaceuticals-17-01041-s001.zip › Figure S1-14_Kin_0.18_Nain_0.03.gif]

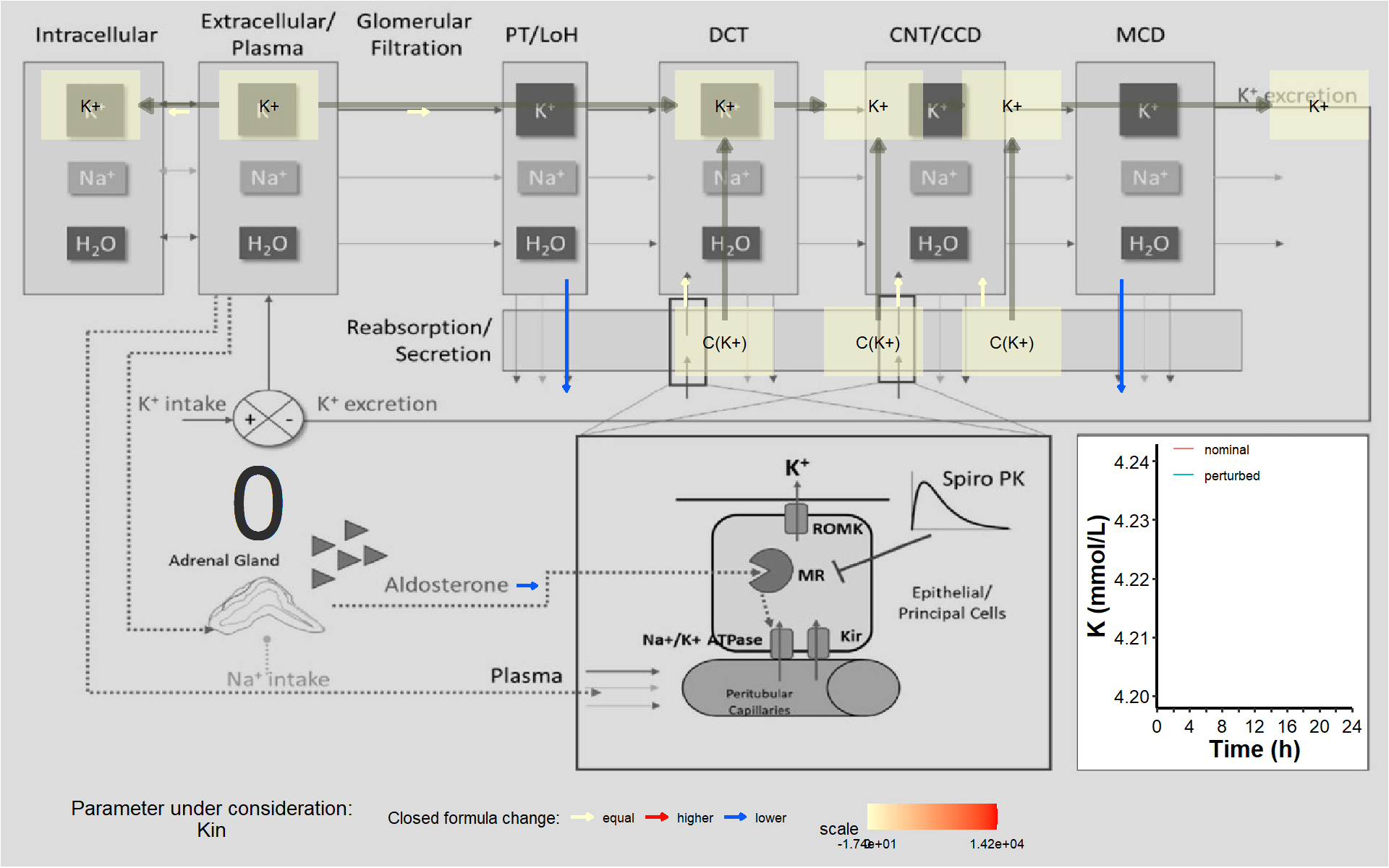

Supplement: Supplementary file 1 [file pharmaceuticals-17-01041-s001.zip › Figure S1-15_Kin_0.18_Nain_0.17.gif]

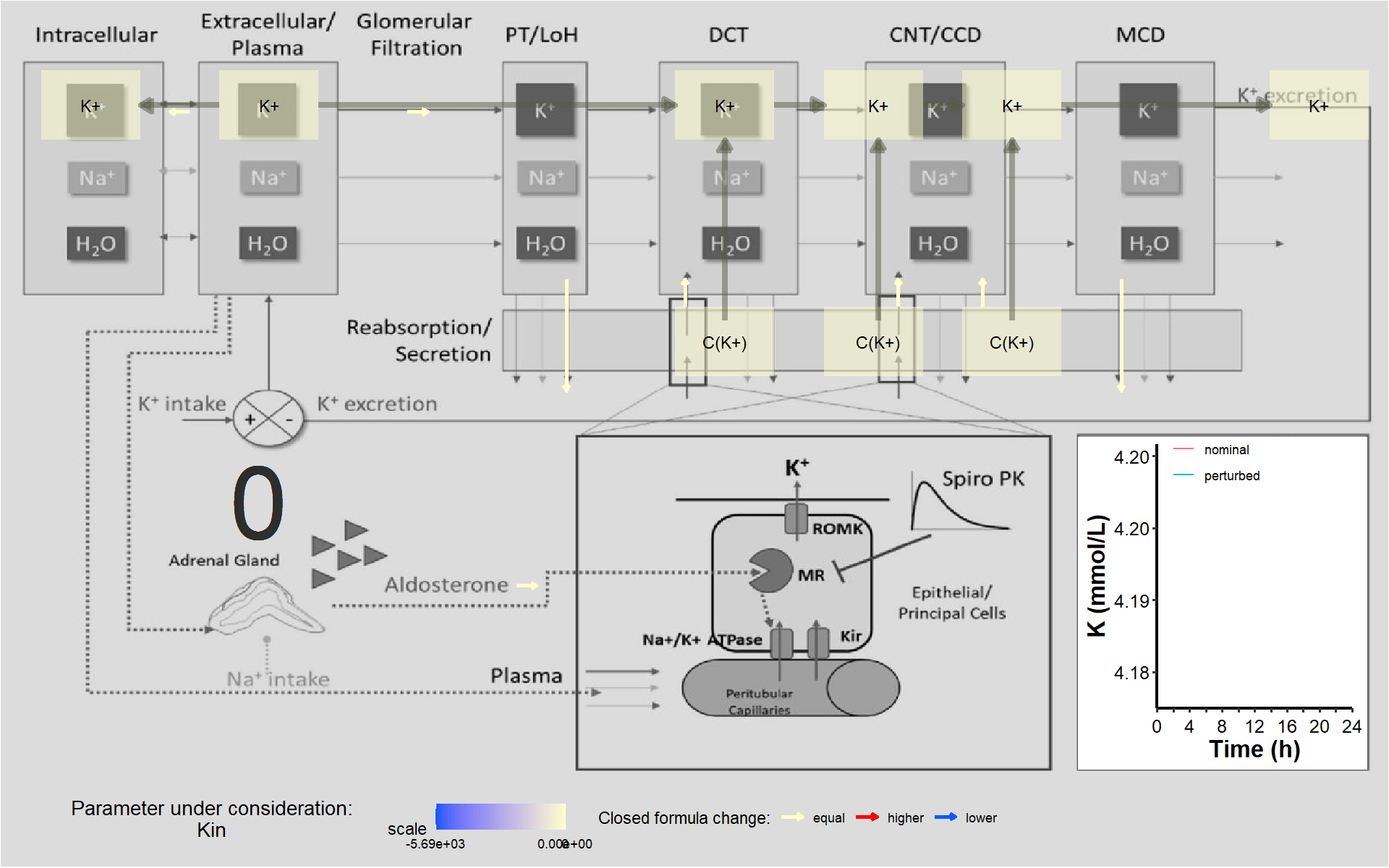

Supplement: Supplementary file 1 [file pharmaceuticals-17-01041-s001.zip › Figure S1-1_Kin_0.04.gif]

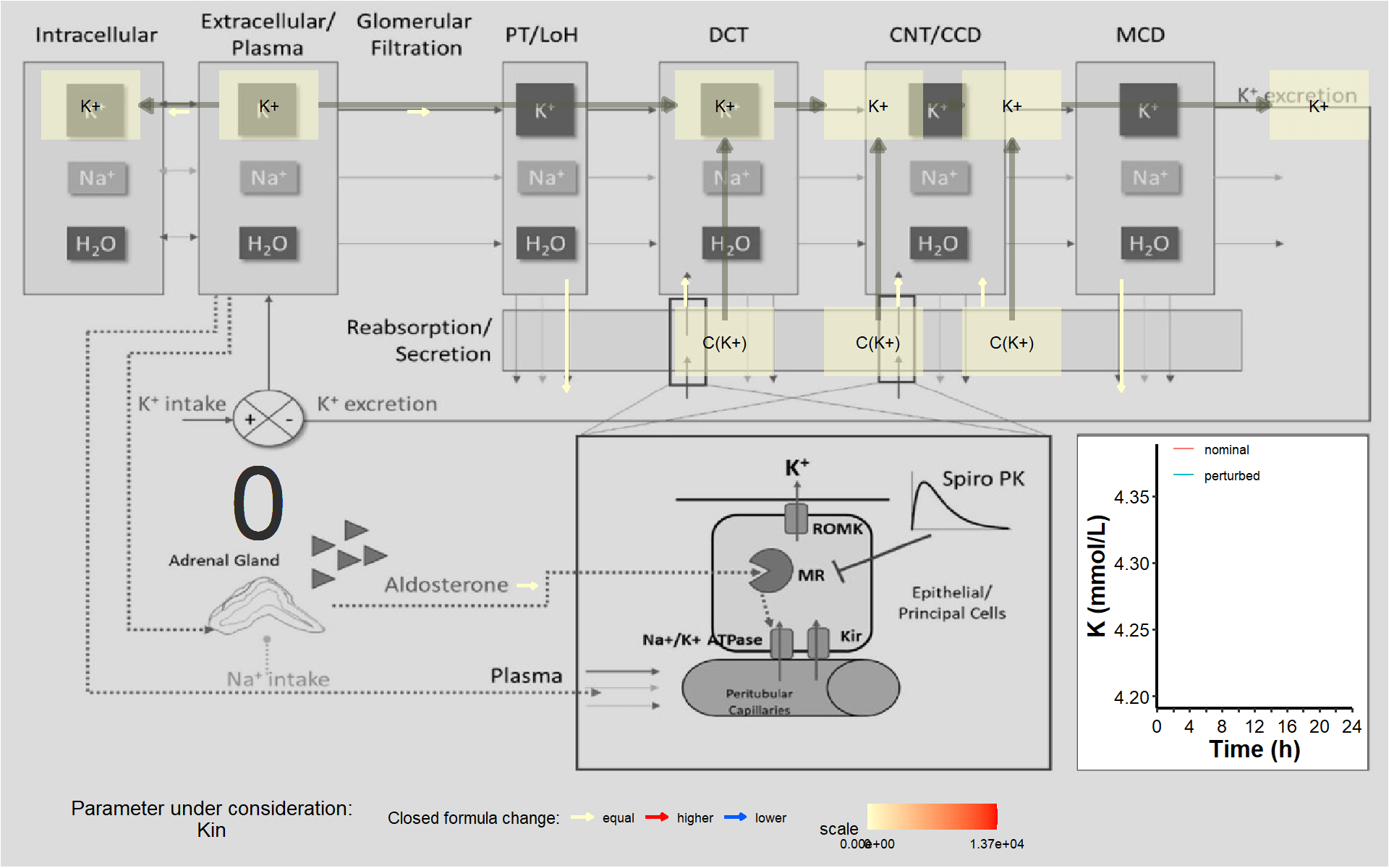

Supplement: Supplementary file 1 [file pharmaceuticals-17-01041-s001.zip › Figure S1-2_Kin_0.18.gif]

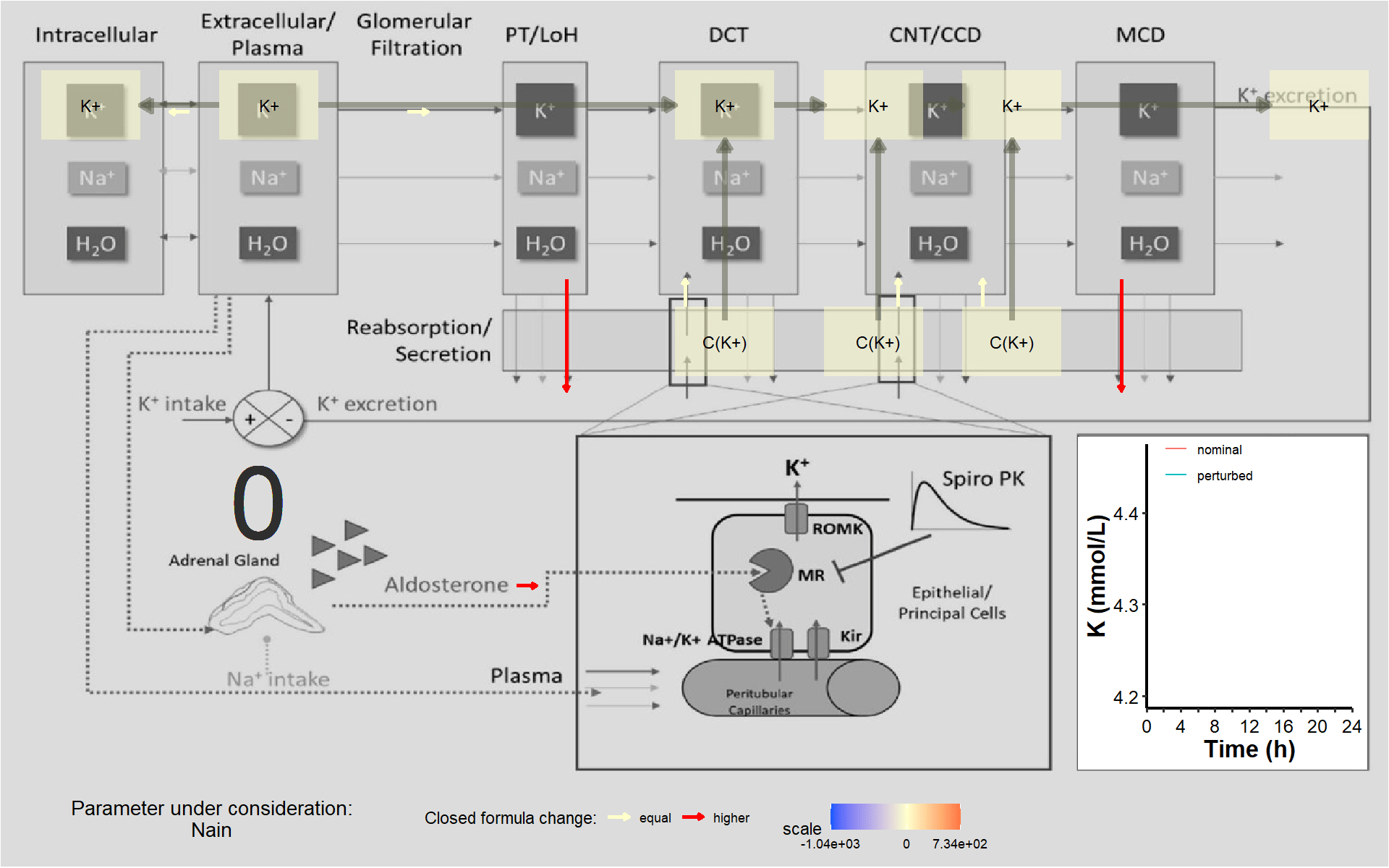

Supplement: Supplementary file 1 [file pharmaceuticals-17-01041-s001.zip › Figure S1-3_Nain_0.03.gif]

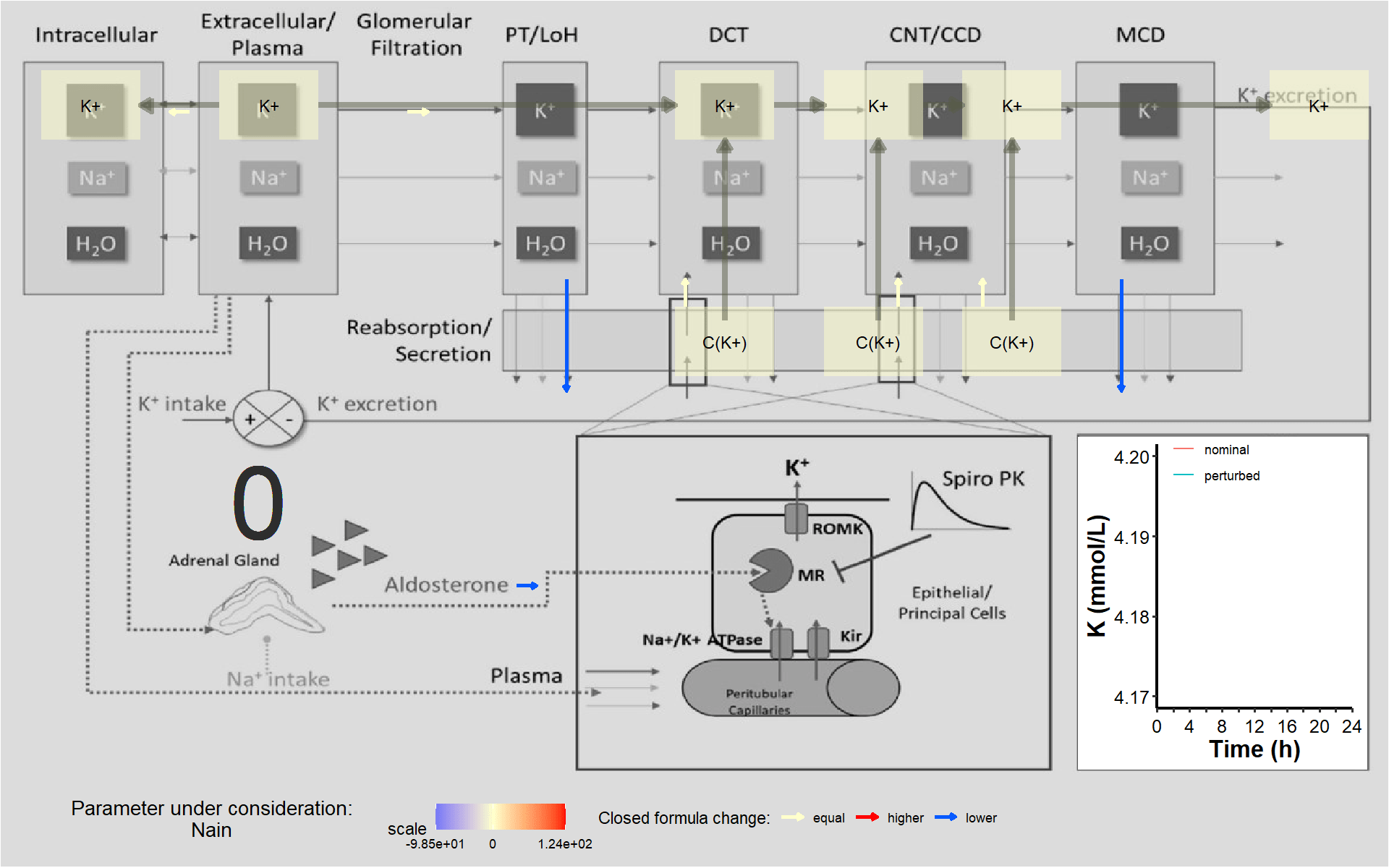

Supplement: Supplementary file 1 [file pharmaceuticals-17-01041-s001.zip › Figure S1-4_Nain_0.17.gif]

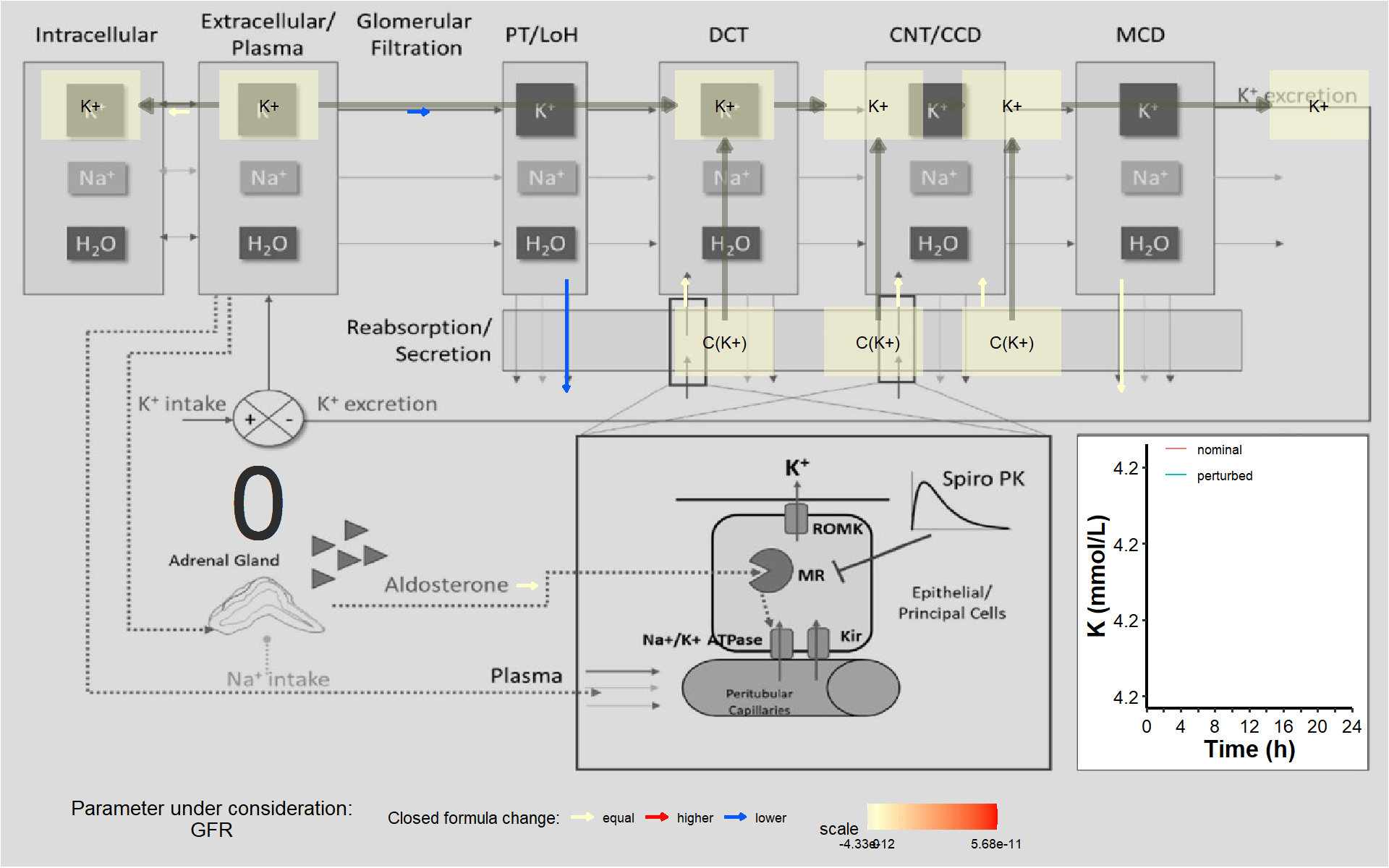

Supplement: Supplementary file 1 [file pharmaceuticals-17-01041-s001.zip › Figure S1-5_GFR_30.gif]

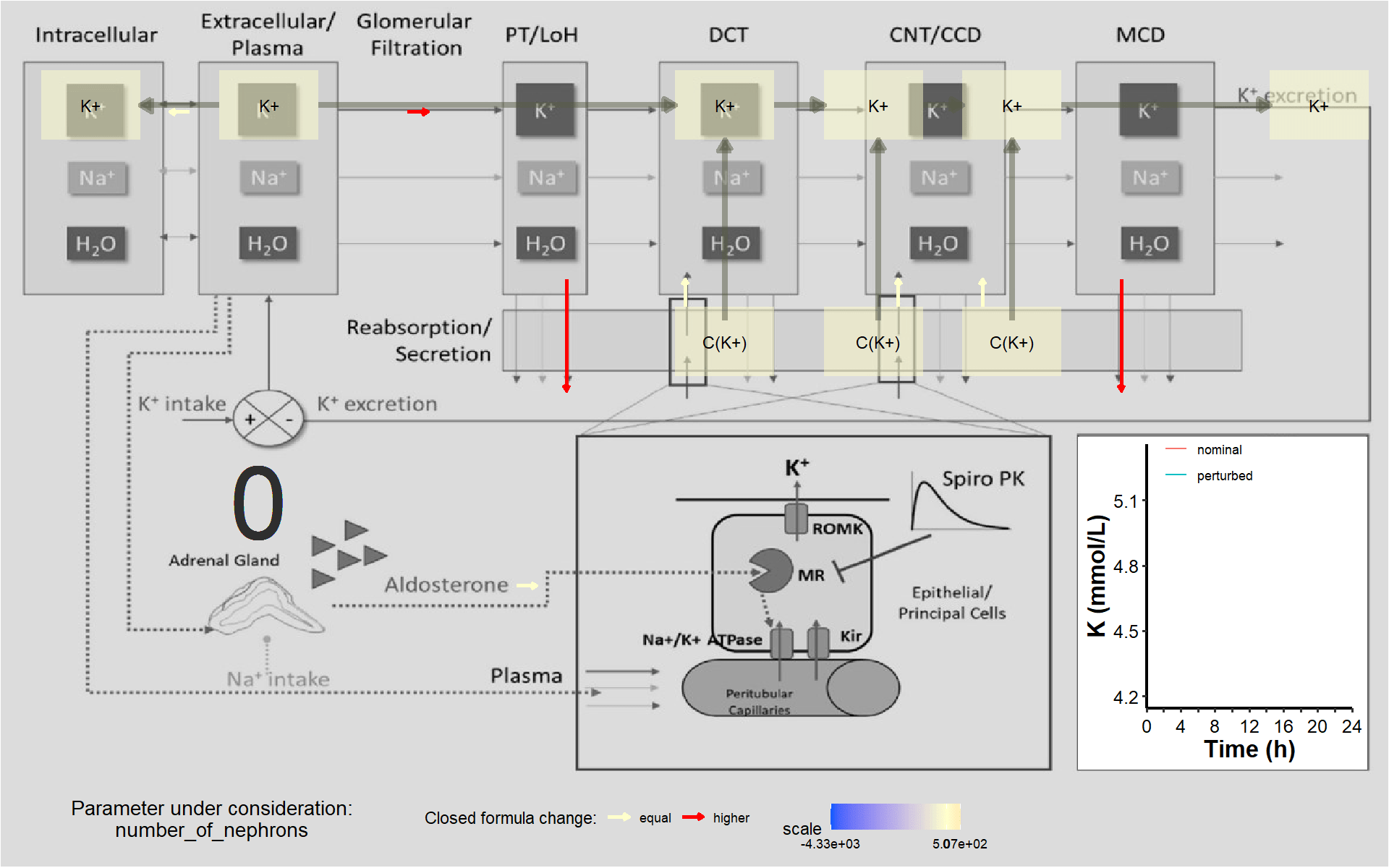

Supplement: Supplementary file 1 [file pharmaceuticals-17-01041-s001.zip › Figure S1-6_number_of_nephrons_20000.gif]

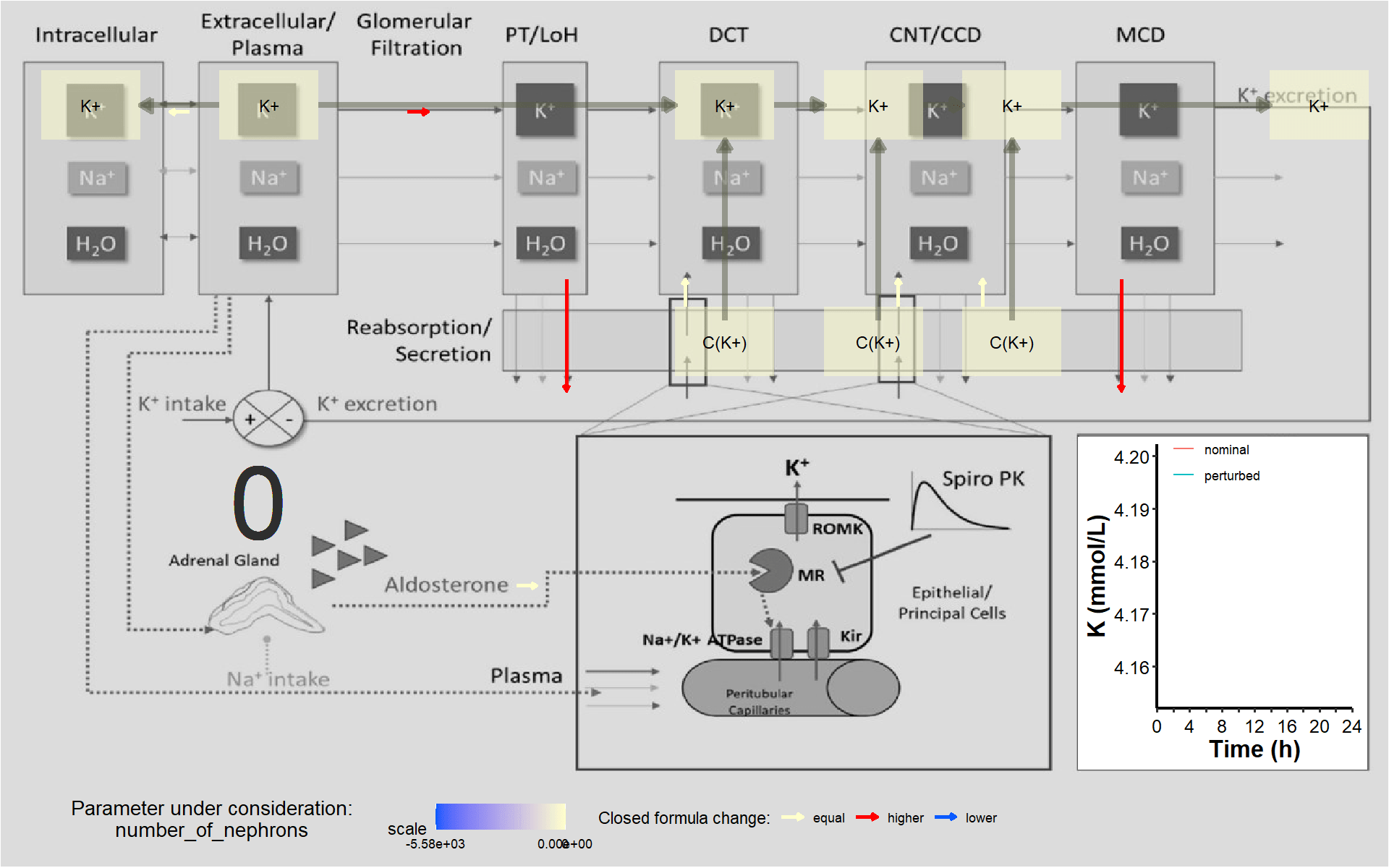

Supplement: Supplementary file 1 [file pharmaceuticals-17-01041-s001.zip › Figure S1-8_number_of_nephrons_20000_Kin_0.04.gif]

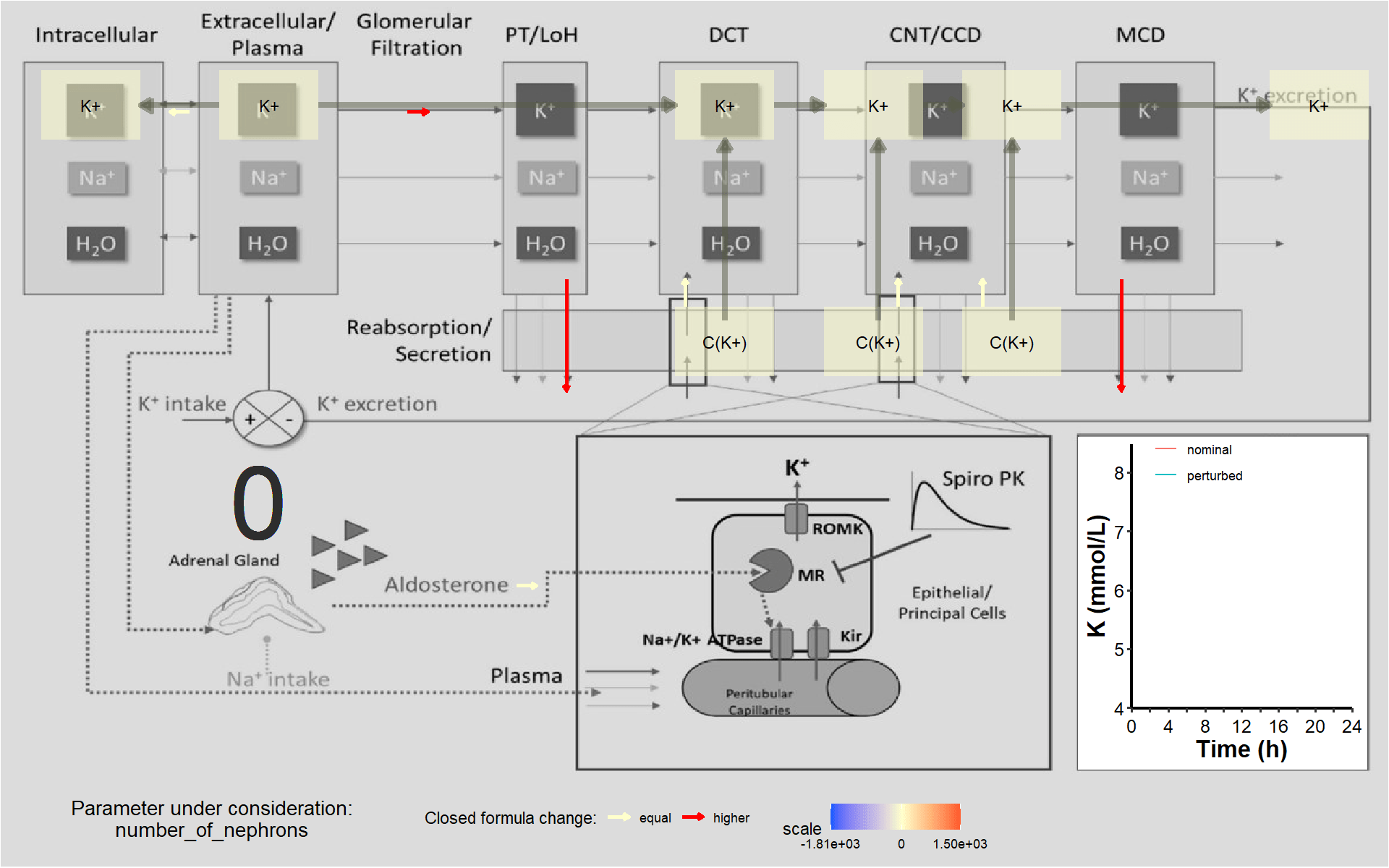

Supplement: Supplementary file 1 [file pharmaceuticals-17-01041-s001.zip › Figure S1-9_number_of_nephrons_20000_Kin_0.18.gif]
